# Exploiting members of the BAHD acyltransferase family to synthesize multiple hydroxycinnamate and benzoate conjugates in yeast

**DOI:** 10.1186/s12934-016-0593-5

**Published:** 2016-11-21

**Authors:** Aymerick Eudes, Maxence Mouille, David S. Robinson, Veronica T. Benites, George Wang, Lucien Roux, Yi-Lin Tsai, Edward E. K. Baidoo, Tsan-Yu Chiu, Joshua L. Heazlewood, Henrik V. Scheller, Aindrila Mukhopadhyay, Jay D. Keasling, Samuel Deutsch, Dominique Loqué

**Affiliations:** 1 Joint BioEnergy Institute, EmeryStation East, 5885 Hollis St., 4th Floor, Emeryville, CA 94608 USA; 2 Biological Systems & Engineering Division, Lawrence Berkeley National Laboratory, 1 Cyclotron Road, Berkeley, CA 94720 USA; 3 Joint Genome Institute, Walnut Creek, CA 94598 USA; 4 Graduate Program, San Francisco State University, San Francisco, CA 94132 USA; 5 Master Program, Ecole Polytechnique Fédérale de Lausanne, 1015 Lausanne, Switzerland; 6 School of BioSciences, The University of Melbourne, Melbourne, VIC 3010 Australia; 7 Department of Chemical & Biomolecular Engineering and Department of Bioengineering, University of California, Berkeley, CA 94720 USA; 8 Novo Nordisk Foundation Center for Biosustainability, Technical University of Denmark, Kogle Alle´, 2970 Hørsholm, Denmark; 9 CNRS, UMR5240, Microbiologie, Adaptation et Pathogénie, Université Claude Bernard Lyon 1, INSA de Lyon, 10 rue Raphaël Dubois, 69622 Villeurbanne, France

**Keywords:** Yeast, BAHD, Antioxidant, Therapeutics, Flavors and fragrances, CAPE

## Abstract

**Background:**

BAHD acyltransferases, named after the first four biochemically characterized enzymes of the group, are plant-specific enzymes that catalyze the transfer of coenzyme A-activated donors onto various acceptor molecules. They are responsible for the synthesis in plants of a myriad of secondary metabolites, some of which are beneficial for humans either as therapeutics or as specialty chemicals such as flavors and fragrances. The production of pharmaceutical, nutraceutical and commodity chemicals using engineered microbes is an alternative, green route to energy-intensive chemical syntheses that consume petroleum-based precursors. However, identification of appropriate enzymes and validation of their functional expression in heterologous hosts is a prerequisite for the design and implementation of metabolic pathways in microbes for the synthesis of such target chemicals.

**Results:**

For the synthesis of valuable metabolites in the yeast *Saccharomyces cerevisiae*, we selected BAHD acyltransferases based on their preferred donor and acceptor substrates. In particular, BAHDs that use hydroxycinnamoyl-CoAs and/or benzoyl-CoA as donors were targeted because a large number of molecules beneficial to humans belong to this family of hydroxycinnamate and benzoate conjugates. The selected BAHD coding sequences were synthesized and cloned individually on a vector containing the Arabidopsis gene *At4CL5,* which encodes a promiscuous 4-coumarate:CoA ligase active on hydroxycinnamates and benzoates. The various *S. cerevisiae* strains obtained for co-expression of At4CL5 with the different BAHDs effectively produced a wide array of valuable hydroxycinnamate and benzoate conjugates upon addition of adequate combinations of donors and acceptor molecules. In particular, we report here for the first time the production in yeast of rosmarinic acid and its derivatives, quinate hydroxycinnamate esters such as chlorogenic acid, and glycerol hydroxycinnamate esters. Similarly, we achieved for the first time the microbial production of polyamine hydroxycinnamate amides; monolignol, malate and fatty alcohol hydroxycinnamate esters; tropane alkaloids; and benzoate/caffeate alcohol esters. In some instances, the additional expression of *Flavobacterium johnsoniae* tyrosine ammonia-lyase (FjTAL) allowed the synthesis of *p*-coumarate conjugates and eliminated the need to supplement the culture media with 4-hydroxycinnamate.

**Conclusion:**

We demonstrate in this study the effectiveness of expressing members of the plant BAHD acyltransferase family in yeast for the synthesis of numerous valuable hydroxycinnamate and benzoate conjugates.

**Electronic supplementary material:**

The online version of this article (doi:10.1186/s12934-016-0593-5) contains supplementary material, which is available to authorized users.

## Background

Chemical synthesis from non-renewable resources is the route most employed for production of chemicals used in both the pharmaceutical and flavor and fragrance industries. Several alternatives have emerged to limit the use of petroleum-based chemicals and to develop environmentally friendly methods through decreasing solvent utilization and reducing the carbon footprint of manufacturing processes. An example of such an alternative is biological synthesis via the use of engineered microbes for the production of fine and specialty chemicals. Because these processes consume renewable lignocellulosic biomass as a carbon source, they have reduced need for toxic chemicals and also offers consistent quality, scalability, simple extraction procedures and the potential for higher synthesis efficiency [1, 2]. In addition, biological synthesis could expand the chemical diversity of natural products, the structural complexity of which is sometimes challenging to achieve using multistep chemical synthesis [3]. In this area, the industrial microorganism *S. cerevisiae* is a powerful host platform for the biosynthesis of plant secondary metabolites such as beta-carotene, amorphadiene, valencene, casbene, cubebol, linalool, patchoulol, resveratrol and vanillin. This is due to its food-grade status, its advantages in the expression of complex metabolic pathways, extensive knowledge regarding its use in large-scale production, the availability of genetic tools, and its biodiversity [4].

**Fig. 1:**
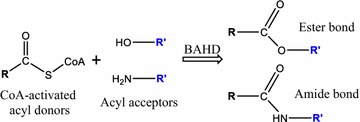
Mechanism of acylation catalyzed by BAHD acyltransferases. Acyl donors are activated upon esterification of their carboxylic group (COOH) with coenzyme A (CoA). BAHD acyltransferases using 4-hydroxycinnamoyl-CoAs (R = 4-hydroxystyrene) and benzoyl-CoAs (R = benzene) as donors were used for this study. R’ = BAHD acceptor

BAHD acyltransferases are plant enzymes that catalyze the transfer of coenzyme A-activated donors—which include acetyl-CoA, *β*-phenylalanine-CoA, malonyl-CoA, tiglyl-CoA, anthraniloyl-CoA, benzoyl-CoA and (hydroxy)cinnamoyl-CoA—onto several types of acceptor molecules [5]. BAHDs catalyze *O*- or *N*-acylation of acceptors by acylating their hydroxyl (OH) or amine (NH_2_) groups, respectively, with CoA donors (Fig. 1). In plants, these enzymes participate in the synthesis of a wide range of polymers and secondary metabolites such as cutin, suberin, lignin, volatiles, pigments and defense compounds [5]. Furthermore, several BAHD acyltransferases catalyze the formation of metabolites beneficial for human health, which has prompted their use as biocatalysts in engineered microbes. For example, engineered *Escherichia coli* strains have been developed to express BAHDs such as hydroxycinnamoyl-CoA:quinate transferases (HQT) for the synthesis of the antioxidant chlorogenic acid [6, 7]; hydroxycinnamoyl-CoA:hydroxyphenyllactate transferases for the production of rosmarinic acid [8–10]; hydroxycinnamoyl-CoA:glycerol transferase for the synthesis of the water-soluble antioxidants hydroxycinnamate glycerol esters [11] and hydroxycinnamoyl/benzoyl-CoA:anthranilate transferase (HCBT) for the production of therapeutic benzoyl and hydroxycinnamoyl anthranilates [12]. To our knowledge, the use of yeast strains engineered for the expression of BAHD acyltransferases has not been reported, except for the synthesis of hydroxycinnamoyl anthranilates using either HCBT or promiscuous hydroxycinnamoyl-CoA:shikimate transferases [13–16].

Hydroxycinnamic acids such as *p*-coumaric acid, caffeic acid, ferulic acid and sinapic acid possess antioxidant properties, antimicrobial activities and have been implicated in the prevention of cardiovascular diseases and cancer [17]. Importantly, the pharmacokinetics and bioavailability of hydroxycinnamic acids, as well as their antimicrobial activity, are highly dependent on their chemical structures and the types of molecules to which they are conjugated [17, 18]. Moreover, certain benzoate conjugates are also used for their health benefits and therapeutic effects [19, 20], whereas benzoate alcohol esters are widely used as flavoring agents in the food and cosmetic industries [21, 22].

In this study, we investigated the potential of 10 members of the BAHD acyltransferase family for the production of diverse hydroxycinnamate and benzoate conjugates in *S. cerevisiae* after feeding with compatible acceptor molecules. These enzymes were selected based on their capacity to use hydroxycinnamoyl-CoAs and/or benzoyl-CoAs as donor molecules and their affinity for structurally divergent acceptors. We have previously reported on a two-gene strategy for co-expression in *S. cerevisiae* of Arabidopsis (*Arabidopsis thaliana*) 4-coumarate:CoA ligase 5 (4CL5) and HCBT for the synthesis of hydroxycinnamoyl anthranilates upon feeding with hydroxycinnamates and anthranilate [13]. We also recently showed evidence that 4CL5 can accept benzoic acid as a substrate, which enabled the production of benzoyl anthranilates when co-expressed with HCBT [14]. Here, taking advantage of 4CL5 substrate’s promiscuity, several BAHD acyltransferases were selected and co-expressed with 4CL5 in an attempt to synthesize in yeast novel hydroxycinnamate and benzoate esters and amides. Furthermore, although the yields of product formation were typically two to fivefold lower compared to those achieved with *p*-coumarate feedings, additional expression of tyrosine ammonia-lyase from *Flavobacterium johnsoniae* (FjTAL), which converts tyrosine into *p*-coumarate [23], enabled the synthesis of *p*-coumarate esters and amides directly from the endogenous tyrosine pool.

## Results and discussion

### Synthesis of rosmarinic acid and analogues

**Fig. 2:**
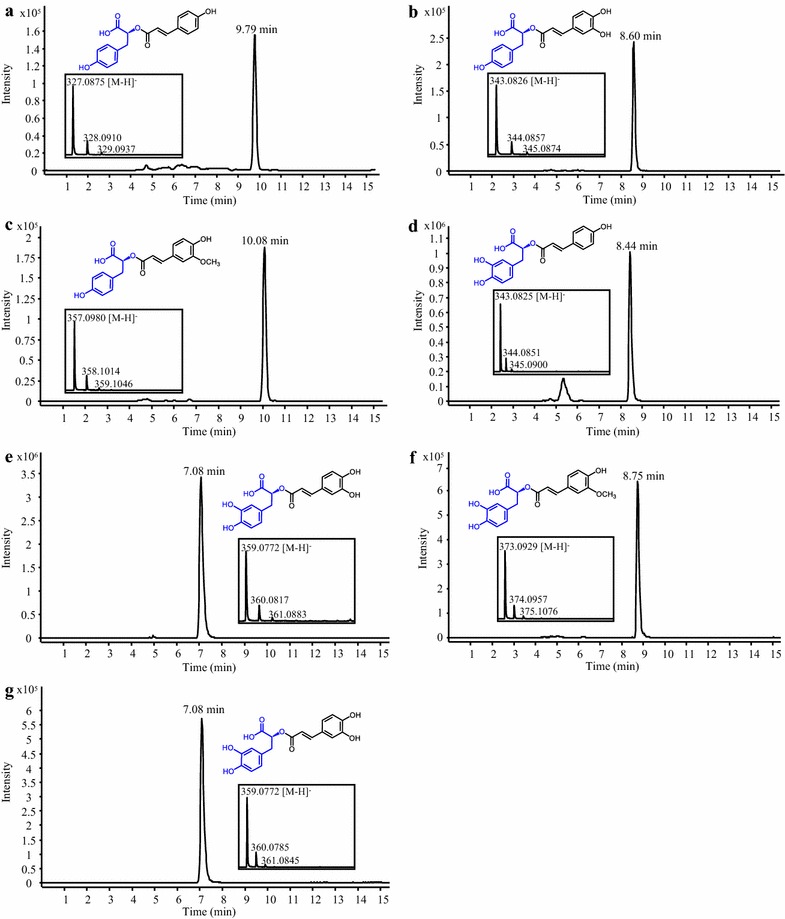
Production of rosmarinic acid and analogues in yeast. Representative LC–MS chromatograms obtained from analysis of the culture medium of a *S. cerevisiae* strain expressing 4CL5 and LaAT1 are shown. The strain was fed with 4-hydroxyphenyllactate and *p*-coumarate, caffeate and ferulate for the synthesis of *p*-coumaroyl 4′-hydroxyphenyllactate (**a**), caffeoyl 4′-hydroxyphenyllactate (**b**) and feruloyl 4′-hydroxyphenyllactate (**c**), respectively. Feeding the strain with 3,4-dihydroxyphenyllactate and *p*-coumarate, caffeate and ferulate allowed the synthesis of *p*-coumaroyl 3′,4′-dihydroxyphenyllactate (**d**), rosmarinic acid (**e**) and feruloyl 3′,4′-dihydroxyphenyllactate (**f)**, respectively. The LC–MS chromatogram of a solution of authentic rosmarinic acid is also shown (**g**)

Rosmarinic acid (RA), a caffeate ester of 3,4-dihydroxyphenyllactate, and its analogues, are strong antioxidants with several beneficial properties for human health [24]. The RA synthase LaAT1 from lavender (*Lavandula angustifolia*), a member of the BAHD family, was selected for expression in yeast because of its substrate promiscuity towards various donors and acceptors [25]. A *S. cerevisiae* strain co-expressing 4CL5 and LaAT1 was generated. Growing this strain in the presence of 4-hydroxyphenyllactate (LaAT1’s preferred acceptor), *p*-coumarate, caffeate and ferulate resulted in the production of *p*-coumaroyl 4′-hydroxyphenyllactate, caffeoyl 4′-hydroxyphenyllactate (isorinic acid) and feruloyl 4′-hydroxyphenyllactate, respectively (Fig. 2a–c). Similarly, feeding the strain with 3,4-dihydroxyphenyllactate as the acceptor and *p*-coumarate, caffeate and ferulate as donors resulted in the production of *p*-coumaroyl 3′,4′-dihydroxyphenyllactate, caffeoyl 3′,4′-dihydroxyphenyllactate (RA) and feruloyl 3′,4′-dihydroxyphenyllactate, respectively (Fig. 2d–f). Quantification of RA using an authentic standard showed that the culture medium contained 429 ± 81 µM (or 154 mg/L) (Fig. 2g). None of these products were observed in the culture medium of a control strain expressing 4CL5 alone and fed with the same precursors (data not shown). To our knowledge, this is the first report of RA biosynthesis in yeast. Moreover, considering the substrate promiscuity of LaAT1 [25], additional RA analogues would likely be produced by supplying the culture medium with alternative donors and acceptors. Such substrate flexibility has been explored for RA synthase from *Coleus blumei* and allowed biosynthesis of 13 RA analogues in *E. coli* [10]. Heterelogous pathways for the synthesis of the two acceptors, 4-hydroxyphenyllactate and 3,4-dihydroxyphenyllactate, from an inexpensive renewable carbon source has already been demonstrated in *E. coli* [8, 9] and could be implemented in yeast for sustainable and economical biosynthesis. We also demonstrated that expression of tyrosine ammonia-lyase (FjTAL) in addition to 4CL5 and LaAT1 results in the production of *p*-coumaroyl 4′-hydroxyphenyllactate when the FjTAL-4CL5-LaAT1 strain is fed with only 4-hydroxyphenyllactate (Additional file 1: Figure S1A). This result shows a potential in yeast for producing hydroxycinnamates more sustainably and directly by using endogenous tyrosine as a precursor pool.

### Synthesis of chlorogenic acid

Caffeoyl quinate (chlorogenic acid) and its derivatives have several beneficial properties for human health [6, 7, 26]. NtHQT, a BAHD enzyme from tobacco (*Nicotiana tabacum*), was selected for the synthesis of coumaroyl quinate and chlorogenic acid in *S. cerevisiae* because it uses both *p*-coumaroyl-CoA and caffeoyl-CoA as donors and quinate as its acceptor [27]. We confirmed that feeding a strain expressing 4CL5 and NtHQT with quinate and *p*-coumarate or caffeate resulted in the production of the two target metabolites, namely coumaroyl quinate and chlorogenic acid (Additional file 1: Figure S2). The subsequent quantification of chlorogenic acid using an authentic standard showed that the culture medium contained 1.43 ± 0.21 µM (or 506 µg/L). To our knowledge, this is the first example of chlorogenic acid biosynthesis in yeast. Because of the conservation of the shikimate pathway between bacteria and yeast, engineering strategies for accumulation of quinate described in *E. coli* [28] could be easily transferred to our 4CL5-NtHQT yeast strain to achieve chlorogenic acid synthesis without feeding quinate.

### Synthesis of glycerol hydroxycinnamates

**Fig. 3:**
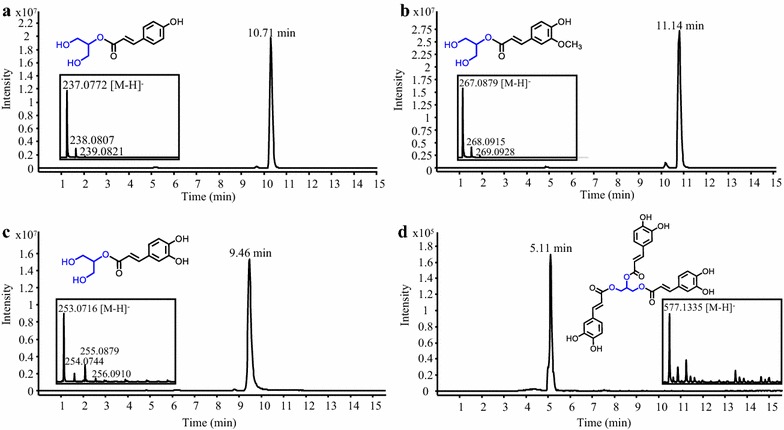
Production of glycerol hydroxycinnamates in yeast. Representative LC–MS chromatograms obtained from analysis of the culture medium of a *S. cerevisiae* strain expressing 4CL5 and OsHCT4 are shown. The strain was fed with glycerol and *p*-coumarate, ferulate and caffeate for the synthesis of *p*-coumaroyl glycerol (**a**), feruloyl glycerol (**b**) and caffeoyl glycerol (**c**), respectively. Structures corresponding to 2-*O*-hydroxycinnamoyl-glycerides are represented as previously determined [11]. A compound with a mass matching that of 1,2,3-tricaffeoyl glycerol was also detected in the case of caffeate feedings (**d**)

Glycerol hydroxycinnamate esters have higher water solubility than non-conjugated hydroxycinnamates and several of these esters, such as feruloyl glycerol conjugates, have antioxidant capacities and neuroprotective effects [29, 30]. Hydroxycinnamoyl-CoA:glycerol transferase OsHCT4 from rice (*Oryza sativa*) was selected for the synthesis of hydroxycinnamoyl glycerol conjugates in yeast [11]. Because OsHCT4 uses *p*-coumaroyl-CoA, feruloyl-CoA and caffeoyl-CoA as donors, a strain co-expressing 4CL5 and OsHCT4 was generated and fed with glycerol and either *p*-coumarate, ferulate or caffeate. For these cultures, analysis of the medium revealed the presence of *p*-coumaroyl glycerol, feruloyl glycerol and caffeoyl glycerol, respectively (Fig. 3a–c). Interestingly, and only in the case of caffeate feeding, a compound with a m/z value matching that of trisubstituted glycerol (i.e., 1,2,3-tricaffeoyl glycerol) was also detected (Fig. 3d). None of these products were observed in the culture medium of a strain expressing 4CL5 alone and fed with the same precursors (data not shown). These experiments are the first example of synthesis of hydroxycinnamate glycerol conjugates in yeast. Finally, using its endogenous glycerol pool, a strain expressing FjTAL, 4CL5 and OsHCT4 produced *p*-coumaroyl glycerol without the addition of any precursors to the culture medium (Additional file 1: Figure S1B).

### Synthesis of polyamine hydroxycinnamates

**Fig. 4:**
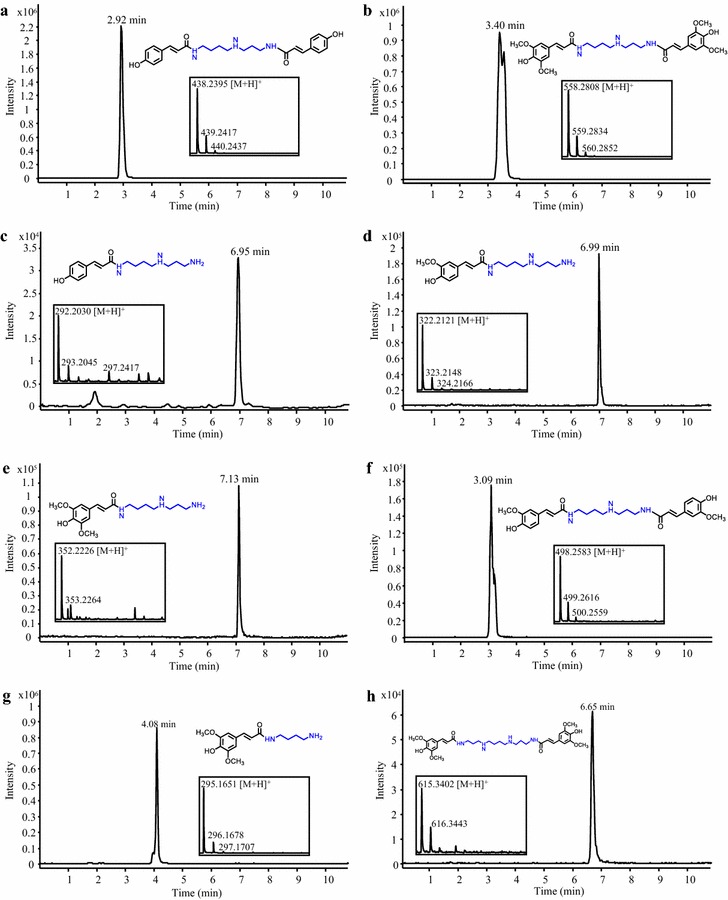
Production of polyamine hydroxycinnamates in yeast. Representative LC–MS chromatograms obtained from analysis of the culture medium of *S. cerevisiae* strains expressing 4CL5 and AtSCT (**a**, **c**–**f**) or 4CL5 and AtSDT (**b**, **g**, **h**) are shown. The 4CL5-AtSCT strain was fed with spermidine and *p*-coumarate, ferulate or sinapate for the synthesis of dicoumaroyl spermidine (**a**) *p*-coumaroyl spermidine (**c**), feruloyl spermidine (**d**), sinapoyl spermidine (**e**) and diferuloyl spermidine (**f**). The 4CL5-AtSDT strain was fed with sinapate and spermidine, putrescine and spermine for the synthesis of disinapoyl spermidine (**b**) sinapoyl putrescine (**g**) and disinapoyl spermine (**h**), respectively. Structures in **a** and **b** are represented as previously determined in [39] whereas other structures are arbitrarily shown with substitutions of the polyamines on *N* ^1^, *N* ^8^ (**c**–**g**) and *N* ^1^, *N* ^12^ (**h**)

Multiple studies have reported on the beneficial properties of hydroxycinnamate amides of polyamines. For example, spermidine hydroxycinnamate conjugates have antioxidant [31, 32], antibacterial [33], antiviral [34], insecticidal [35] and antiparasitic [36] activities. Other studies have demonstrated the inhibitory activity of polysubstituted *p*-coumaroyl polyamines on brain transporters for which a genetic polymorphism is associated with psychological disorders [37, 38]. For this experiment, we selected the BAHDs *p*-coumaroyl-CoA:spermidine (AtSCT) and sinapoyl-CoA:spermidine (AtSDT) transferases from Arabidopsis [39], and *p*-coumaroyl-CoA:agmatine transferase (HvACT) from barley (*Hordeum vulgare*) [40]. Consistent with the properties of both AtSCT and AtSDT to catalyze the transfer of two hydroxycinnamate moieties onto spermidine [39], feeding with spermidine and *p*-coumarate or sinapate strains co-expressing 4CL5 with AtSCT or AtSDT resulted in the synthesis of dicoumaroyl and disinapoyl spermidine conjugates, respectively (Fig. 4a, b). In particular, a single new liquid chromatography-mass spectrometry (LC–MS) peak with a m/z value corresponding to 438.2395 [M + H]^+^ was detected in the medium of the 4CL5-AtSCT strain (Fig. 4a), suggesting the presence of *N* ^1^,*N* ^8^-dicoumaroyl spermidine as previously observed in vitro [39]. In the case of the 4CL5-AtSDT strain, LC–MS analysis of the culture medium showed a double LC–MS peak with a m/z value corresponding to 558.2808 [M + H]^+^ (Fig. 4b), suggesting the presence of another disinapoyl spermidine isomer (i.e. *N* ^1^,*N* ^4^-disinapoyl spermidine or *N* ^4^,*N* ^8^-disinapoyl spermidine) in addition to the previously characterized AtSDT product, *N* ^1^,*N* ^8^-disinapoyl spermidine [39]. The presence of a monosubstituted spermidine (i.e., *p*-coumaroyl spermidine) was also observed in the culture medium of the 4CL5-AtSCT strain but not in that of the 4CL5-AtSDT strain (Fig. 4c). Experiments exploring the substrate promiscuity of AtSDT and AtSCT were conducted to produce additional polyamine hydroxycinnamates. Feeding the 4CL5-AtSCT strain with spermidine and ferulate or sinapate successfully enabled the biosynthesis of feruloyl spermidine and sinapoyl spermidine, respectively (Fig. 4d, e). Moreover, a double LC–MS peak with a m/z value matching that of diferuloyl spermidine was detected when ferulate was supplied (Fig. 4f), but the exact identities of the two isomers was not determined. Next, feeding the 4CL5-AtSDT strain with sinapate and putrescine or spermine resulted in the synthesis of sinapoyl putrescine and disinapoyl spermine, respectively (Fig. 4g, h). These results show that the substrate flexibility of the two enzymes toward both donors and acceptors can be exploited for the synthesis of diverse polyamine hydroxycinnamates. This is the first report of biological synthesis of polyamine hydroxycinnamates in microbes, and our results show the potential for the production of disubstituted polyamine conjugates. We also demonstrated that co-expression of FjTAL with 4CL5 and AtSCT resulted in the production of *N* ^1^,*N* ^8^-dicoumaroyl spermidine without the addition of any precursors to the culture medium (Additional file 1: Figure S1C), providing a potential route to sustainable synthesis of such compounds. This indicates that spermidine pools in *S. cerevisiae* are sufficient for such biosynthetic approaches. In that respect, recently developed engineering strategies for enhancing spermidine content in yeast could be applied to our strain [41]. Lastly, our list of microbially produced polyamine hydroxycinnamates was extended after a strain co-expressing FjTAL, 4CL5 and HvACT and fed with agmatine was able to produce *p*-coumaroyl agmatine (Additional file 1: Figure S1D).

### Synthesis of monolignol hydroxycinnamates

**Fig. 5:**
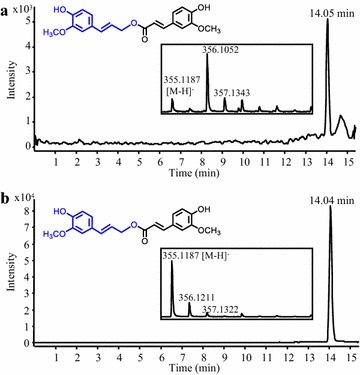
Production of coniferyl ferulate in yeast. A representative LC–MS chromatogram of a hexane extract of isolated *S. cerevisiae* cells expressing 4CL5 and AsFMT and previously grown in the presence of ferulate and coniferyl alcohol is shown (**a**). Identification of intracellular coniferyl ferulate was made by comparison with the LC–MS profile of a solution of authentic coniferyl ferulate (**b**)

Monolignol hydroxycinnamates belong to another class of hydroxycinnamate ester conjugates with beneficial pharmacological properties. For example, coniferyl ferulate has antibacterial activity, antioxidant and anti-Alzheimer properties, and it was also shown to have a potential role in reversing multidrug resistance mechanisms acquired by tumor cells [42–44]. Feruloyl-CoA:monolignol transferase (AsFMT) from Chinese angelica (*Angelica sinensis*) is a BAHD enzyme involved in the synthesis of coniferyl ferulate by coupling feruloyl-CoA to the monolignol coniferyl alcohol [45]. A *S. cerevisiae* strain that co-expressed 4CL5 with AsFMT was generated and grown in the presence of ferulate and coniferyl alcohol for the synthesis of coniferyl ferulate. Interestingly, using LC–MS analysis and an authentic standard for comparison, we observed that coniferyl ferulate was effectively produced by the yeast strain (Fig. 5a–b) but remained exclusively intracellular. This is the first successful attempt at producing coniferyl ferulate in microbes. To facilitate its purification and reduce production costs, it would be useful to evaluate other hosts for their capability to synthesize and excrete this valuable product. In this respect, several strains of *E*. *coli* have been engineered for the production of *p*-coumaryl alcohol via *p*-coumaroyl-CoA and could become suitable hosts [46, 47].

### Synthesis of malate hydroxycinnamates

**Fig. 6:**
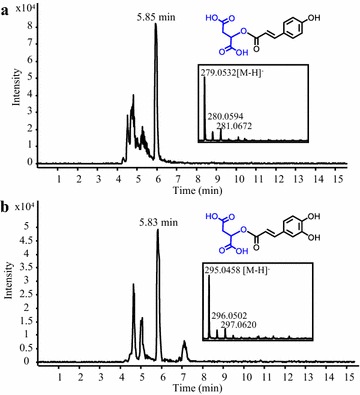
Production of malate hydroxycinnamates in yeast. Representative LC–MS chromatograms obtained from analysis of the culture medium of a *S. cerevisiae* strain expressing 4CL5 and TpHCT2 are shown. The strain was fed with malate and *p*-coumarate or caffeate for the synthesis of *p*-coumaroyl malate (**a**) and phaselic acid (**b**), respectively

Malate hydroxycinnamates are another group of hydroxycinnamate esters with potential beneficial properties for human health, as exemplified by caffeoyl malate (phaselic acid), which possesses anti-inflammatory activities [48]. Hydroxycinnamoyl-CoA:malate transferase from red clover (*Trifolium pratense*) (TpHCT2) was selected for the production of malate hydroxycinnamates in yeast [49]. A *S. cerevisiae* strain co-expressing 4CL5 and TpHCT2 was fed with malate and *p*-coumarate or caffeate. LC–MS analysis of the culture medium showed the presence of peaks corresponding to *p*-coumaroyl malate and phaselic acid, respectively (Fig. 6a, b). These LC–MS peaks were absent from control cultures of a strain expressing 4CL5 alone and fed with the same precursors (data not shown). This is the first example of the production of malate hydroxycinnamates in microbes, and such synthesis could theoretically be achieved sustainably considering existing engineering methods used to obtain high titers of malate in yeast [50]. Concerning the *de novo* synthesis of hydroxycinnamate moieties, we also show that expression of FjTAL in the 4CL5-TpHCT2 strain can sustain the synthesis of *p*-coumaroyl malate without an external supply of *p*-coumarate (Additional file 1: Figure S1E).

### Synthesis of alkyl hydroxycinnamates

**Fig. 7:**
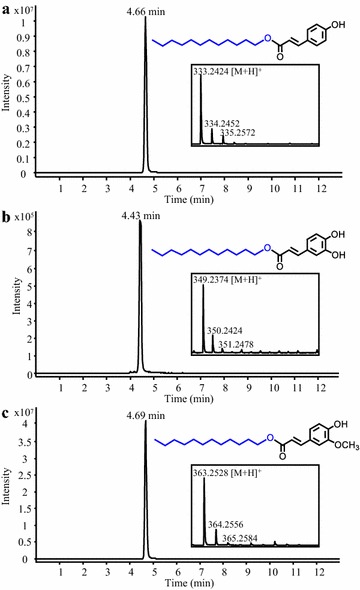
Production of dodecyl hydroxycinnamates in yeast. Representative LC–MS chromatograms obtained from analysis of the culture medium of a *S. cerevisiae* strain expressing 4CL5 and AtHHT3 are shown. The strain was fed with 1-dodecanol and *p*-coumarate, caffeate or ferulate for the synthesis of dodecyl *p*-coumarate (**a**), dodecyl caffeate (**b**) and dodecyl ferulate (**c**), respectively

Alkyl hydroxycinnamates such as hydroxycinnamate esters of fatty alcohols are lipophilic antioxidants that can efficiently inhibit the growth of tumor cells [51, 52]. Hydroxycinnamoyl-CoA:fatty alcohol transferase from Arabidopsis (AtHHT3), which accepts various medium chain fatty alcohols as substrates, was selected for the synthesis of dodecyl hydroxycinnamates [53]. In particular, a *S. cerevisiae* strain co-expressing 4CL5 with AtHHT3 and fed with 1-dodecanol and *p*-coumarate, caffeate or ferulate produced the potent antioxidants dodecyl *p*-coumarate, dodecyl caffetae and dodecyl ferulate, respectively (Fig. 7a–c). These alkyl hydroxycinnamates were not detected in cultures of a control strain expressing 4CL5 alone and fed with hydroxycinnamates and 1-dodecanol (data not shown). The microbial synthesis of alkyl hydroxycinnamates has never been reported and engineering strategies used for the production of fatty alcohols could be leveraged for their *de novo* synthesis [54, 55]. Moreover, using a strain that co-express FjTAL with 4CL5 and AtHHT3, we achieved the production of dodecyl *p*-coumarate by supplying only 1-dodecanol to the culture medium (Additional file 1: Fig. S1F).

### Synthesis of cinnamoyl and benzoyl tropane analogues

**Fig. 8:**
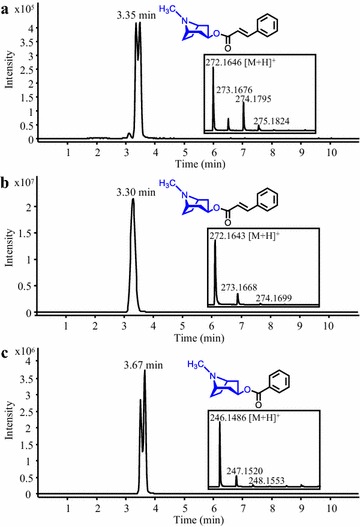
Production of tropane alkaloids in yeast. Representative LC–MS chromatograms obtained from analysis of the culture medium of a *S. cerevisiae* strain expressing 4CL5 and EcCS are shown. The strain was fed with 3β-tropine and cinnamate or benzoate for the synthesis of cinnamoyl 3β-tropine (**a**) and tropacocaine (**c**), respectively. Note the double LC–MS peaks suggesting the presence of two isomers for each product. The LC–MS chromatogram of a solution of authentic cinnamoyl 3β-tropine is also shown (**b**)

Tropane alkaloids, such as cinnamate and benzoate esters of tropane skeletons, are of considerable interest due to their wide range of biological activities, and some are commonly employed in the pharmaceutical industry [56, 57]. Cocaine synthase (EcCS) from coca (*Erythroxylum coca*) catalyzes the transfer of both cinnamoyl-CoA and benzoyl-CoA onto 2-carbomethoxy-3β-tropine for the synthesis of the tropane alkaloids cinnamoylcocaine and cocaine, respectively [58]. Because we previously demonstrated the activity of 4CL5 towards cinnamate and benzoate in yeast [14], and considering that EcCS was also shown to use 3β-tropine as an acceptor in vitro [58], we generated a *S. cerevisiae* strain that co-express 4CL5 and EcCS for the synthesis of the tropane alkaloids cinnamoyl 3β-tropine and benzoyl 3β-tropine (tropacocaine). By comparison with an authentic standard, LC–MS analysis identified cinnamoyl 3β-tropine (9.58 ± 1.06 µM or 2.6 mg/L) in the culture medium of the 4CL5-EcCS strain grown in the presence of cinnamate and 3β-tropine (Fig. 8a, b). Similarly, benzoyl 3β-tropine was detected in the culture medium of the 4CL5-EcCS strain cultivated with the precursors benzoate and 3β-tropine (Fig. 8c). None of these products were detected in control cultures of a strain expressing 4CL5 alone and fed with the same precursors (data not shown). Nevertheless, further investigation will be needed since the detection of double LC–MS peaks strongly suggests the presence of two isomers for each product, possibly due to the isomerization of 3β-tropine in the culture medium. To our knowledge, this is the first report on the synthesis of tropane alkaloids in microbes, and it would be interesting to investigate the substrate flexibility of EcCS for the synthesis of pharmacologically valuable cinnamoyl and benzoyl tropane analogues [20]. As future work, the plant β-oxidative pathway of benzoic acid biosynthesis could be imported into yeast for the synthesis of benzoyl-CoA from phenylalanine [59], as well as the metabolic engineering strategies already established in hairy root culture systems for the synthesis of tropane moieties from arginine [60].

### Synthesis of benzoate alcohol esters and caffeate phenethyl ester (CAPE)

**Fig. 9:**
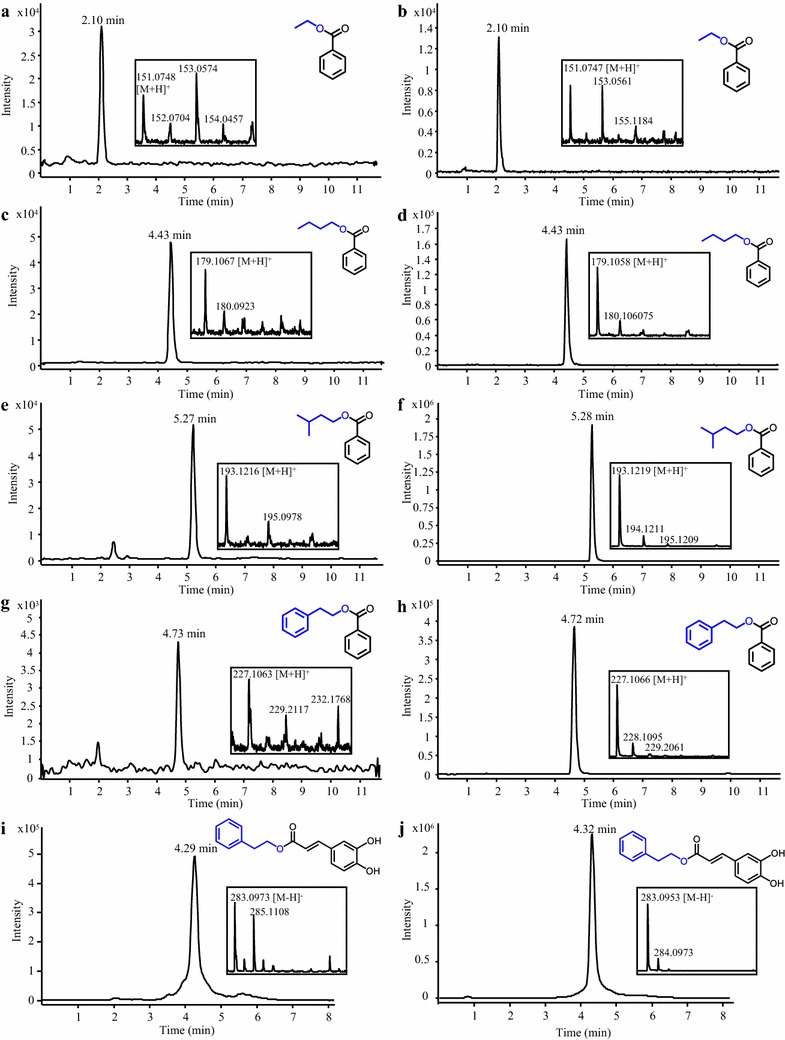
Production of benzoate alcohol esters and CAPE in yeast. Representative LC–MS chromatograms of hexane extracts of culture media of a *S. cerevisiae* strain expressing 4CL5 and BPBT are shown. The strain was fed with benzoate and ethanol, butanol, isopentanol or 2-phenylethanol for the synthesis of ethyl benzoate (**a**), butyl benzoate (**c**), isopentyl benzoate (**e**) and phenethyl benzoate (**g**), respectively. Identification of the different benzoate alcohol esters was made by comparison with the LC–MS profiles of authentic standards (**b**, **d**, **f**, **h**). Similarly, feeding the strain with caffeate and 2-phenylethanol allowed the synthesis of CAPE (**i**) by comparison with the LC–MS profile of an authentic CAPE standard (**j**)

Benzoate alcohol esters are widely used as flavoring agents in foods and cosmetics as well as in hygiene and pharmaceutical products [21, 22]. Benzoyl-CoA:benzyl alcohol/phenylethanol benzoyltransferase (BPBT), a BAHD enzyme from petunia (*Petunia* x *hybrida*), uses benzoyl-CoA as a donor and various alcohols as acceptors for the formation of benzoate alcohol esters [61]. A *S. cerevisiae* strain was generated for the co-expression of 4CL5 and BPBT. Based on the substrate preference of BPBT observed in vitro [61], our 4CL5-BPBT strain was fed with benzoic acid in combination with ethanol, butanol, isopentanol or 2-phenylethanol. By comparison with the elution of authentic standards, LC–MS analysis of the culture media showed the presence of ethyl benzoate (1.39 ± 0.40 µM or 209 µg/L), butyl benzoate (2.65 ± 0.69 µM or 471 µg/L), isopentyl benzoate (2.50 ± 0.50 µM or 481 µg/L) and phenethyl benzoate (7.80 ± 1.73 µM or 1763 µg/L), respectively (Fig. 9a–h). Moreover, caffeate phenethyl ester (CAPE) is a bioactive compound that possesses antimicrobial, antioxidant, anti-inflammatory and cytotoxic properties [62, 63]. Therefore, the capacity of BPBT to accept 2-phenylethanol as a substrate is of particular interest toward the biological synthesis of CAPE and its derivatives, especially because its production using chemical synthesis or its purification from natural sources (e.g., honeybee hives) are low-yielding procedures [64]. Feeding our 4CL5-BPBT strain with caffeate and 2-phenylethanol resulted in successful synthesis of CAPE (1.61 ± 0.23 nM or 457 ng/L), which was identified by LC-MS analysis of the culture medium (Fig. 9i–j). Although several BAHDs acyltransferases similar to BPBT have been shown to transfer benzoyl-CoA onto 2-phenylethanol [65, 66], the use of hydoxycinnamoyl-CoA donors (e.g., caffeoyl-CoA) by these enzymes has never been reported and could enable the biological synthesis of valuable hydroxycinnamate phenethyl esters. Taking advantage of existing engineering strategies that yield high titers of alcohols and 2-phenylethanol should enable the economical synthesis of benzoate alcohol esters and CAPE in yeast [67, 68].

## Conclusions

This study illustrates the potential of using BAHD acyltransferases from plants and *S. cerevisiae* as an expression host for the biological synthesis of valuable molecules that are usually produced from petrochemicals or found at very low concentration in plant extracts. In particular, expression of BAHD acyltransferases that use hydoxycinnamoyl-CoA and benzoyl-CoA allowed the synthesis of more than 30 different compounds which have applications across diverse sectors such as human health products and the flavor and fragrance industry. Exploring the substrate promiscuity of these BAHDs could considerably expand the number of molecules produced. Moreover, the use of BAHDs activity on alternate donors such as acetyl-CoA or malonyl-CoA should be explored for the biosynthesis of additional beneficial products.

## Methods

### Chemicals

The chemicals used for the *S. cerevisiae* feeding are listed in Additional file 1: Table S1. Rosmarinic acid was obtained from Spectrum Chemical Mfg. Corp. (New Brunswick, NJ), chlorogenic acid from Alfa Aesar (Haverhill, MA), ethyl benzoate, butyl benzoate, isopentyl benzoate and caffeate phenetyl ester from TCI America (Portland, OR), cinnamoyl 3β-tropine and phenethyl benzoate from Sigma-Aldrich (St. Louis, MO), and coniferyl ferulate from Biorbyt LLC (Berkeley, CA).

### Strains, plasmids and media

The vectors pDRf1-4CL5-AtSDT, pDRf1-4CL5-AtSCT, pDRf1-4CL5-HvACT, pDRf1-4CL5-OsHCT4, pDRf1-4CL5-AsFMT, pDRf1-4CL5-EcCS, pDRf1-4CL5-TpHCT2, pDRf1-4CL5-LaAT1, pDRf1-4CL5-NtHQT, pDRf1-4CL5-AtHHT3, and pDRf1-4CL5-BPBT were used for co-expression of 4CL5 with the different BAHDs under the control of the constitutive promoters P_*HXT7*_ and P_*PMA1*_, respectively (Table 1). The Gateway-enabled pRS423 vector was used for the expression of FjTAL under the control of the constitutive promoter P_*TDH3*_. The *S. cerevisiae pad1* knockout (*MAT*a *his3*∆*1 leu2*∆*0 met15*∆*0 ura3*∆*0* ∆*pad1*, ATCC 4005833) [69] was transformed using the Frozen-EZ Yeast Transformation II Kit™ (Zymo Research Corporation, Irvine, CA) and selected on solid medium containing yeast nitrogen base (YNB) without amino acids (Difco 291940; Difco, Detroit, MI) supplemented with 3% glucose and 1X dropout-uracil (CSM-ura) or 1X dropout-uracil-histidine (CSM-ura-his) (Sunrise Science Products, San Diego, CA).

**Table 1:** Plasmids used in this study

Plasmid name	Description	Reference
pDRf1-4CL5	*URA3* selectable marker, arabidopsis *4CL5* P_*HXT7*_-*At4CL5*-T_*CYC1*_ (*URA3*, Amp^R^)	[13]
pDRf1-4CL5-GW	*URA3* selectable marker, arabidopsis *4CL5*, gateway cloning cassette P_*HXT7*_-*At4CL5*-T_*CYC1*_-P_*PMA1*_-*attR1*-*Cm* ^*R*^-*ccdB*-*attR2*-T_*ADH1*_ (*URA3*, Amp^R^)	[13]
pDRf1-4CL5-DsRed	*URA3* selectable marker, arabidopsis *4CL5*, DsRed dropout cassette P_*HXT7*_-*At* *4CL5*-T_*CYC1*_-P_*PMA1*_-*DsRed*-T_*ADH1*_ (*URA3*, Amp^R^)	This work
pRS423-GW	*HIS3* selectable marker, Gateway cloning cassette P_*TDH3*_-*attR1*-*Cm* ^*R*^-*ccdB*-*attR2*-T_*CYC1*_ (*HIS3*, Amp^R^)	This work
pRS423-FjTAL	*HIS3* selectable marker, *Flavobacterium johnsoniae TAL* P_*TDH3*_-*FjTAL*-T_*CYC1*_ (*HIS3*, Amp^R^)	This work
pDRf1-4CL5-AsFMT	*URA3* selectable marker, arabidopsis *4CL5*, *Angelica sinensis FMT* P_*HXT7*_-*At4CL5*-T_*CYC1*_-P_*PMA1*_-*AsFMT*-T_*ADH1*_ (*URA3*, Amp^R^)	This work
pDRf1-4CL5-AtHHT3	*URA3* selectable marker, arabidopsis *4CL5*, arabidopsis *HHT3* P_*HXT7*_-*At4CL5*-T_*CYC1*_-P_*PMA1*_-*AtHHT3*-T_*ADH1*_ (*URA3*, Amp^R^)	This work
pDRf1-4CL5-AtSCT	*URA3* selectable marker, arabidopsis *4CL5*, arabidopsis *SCT* P_*HXT7*_-*At4CL5*-T_*CYC1*_-P_*PMA1*_-*AtSCT*-T_*ADH1*_ (*URA3*, Amp^R^)	This work
pDRf1-4CL5-AtSDT	*URA3* selectable marker, arabidopsis *4CL5*, arabidopsis *SDT* P_*HXT7*_-*At4CL5*-T_*CYC1*_-P_*PMA1*_-*AtSDT*-T_*ADH1*_ (*URA3*, Amp^R^)	This work
pDRf1-4CL5-BPBT	*URA3* selectable marker, arabidopsis *4CL5*, *Petunia* x *hybrida BPBT* P_*HXT7*_-*At4CL5*-T_*CYC1*_-P_*PMA1*_-*BPBT*-T_*ADH1*_ (*URA3*, Amp^R^)	This work
pDRf1-4CL5-EcCS	*URA3* selectable marker, arabidopsis *4CL5*, *Erythroxylum coca CS* P_*HXT7*_-*At4CL5*-T_*CYC1*_-P_*PMA1*_-*EcCS*-T_*ADH1*_ (*URA3*, Amp^R^)	This work
pDRf1-4CL5-HvACT	*URA3* selectable marker, arabidopsis *4CL5*, *Hordeum vulgare ACT* P_*HXT7*_-*At4CL5*-T_*CYC1*_-P_*PMA1*_-*HvACT*-T_*ADH1*_ (*URA3*, Amp^R^)	This work
pDRf1-4CL5-NtHQT	*URA3* selectable marker, arabidopsis *4CL5*, *Nicotiana tabacum HQT* P_*HXT7*_-*At4CL5*-T_*CYC1*_-P_*PMA1*_-*NtHQT*-T_*ADH1*_ (*URA3*, Amp^R^)	This work
pDRf1-4CL5-OsHCT4	*URA3* selectable marker, arabidopsis *4CL5*, *Oryza sativa HCT4* P_*HXT7*_-*At4CL5*-T_*CYC1*_-P_*PMA1*_-*OsHCT4*-T_*ADH1*_ (*URA3*, Amp^R^)	This work
pDRf1-4CL5-TpHCT2	*URA3* selectable marker, arabidopsis *4CL5*, *Trifolium pratense TpHCT2* P_*HXT7*_-*At4CL5*-T_*CYC1*_-P_*PMA1*_-*TpHCT2*-T_*ADH1*_ (*URA3*, Amp^R^)	This work

### Construction of plasmids

A description of the plasmids used in this study is provided in Table 1. For the construction of the pDRf1-4CL5-DsRed vector, a red fluorescent protein (DsRed) dropout cassette sequence (kindly provided by Chris Anderson’s lab, UC Berkeley) was synthesized with flanking sequences (Genescript, Piscataway, NJ; Additional file 1: Data S1). It contains at the 5′-end the 18-bp sequence located upstream of the *Eco*RI site in pDRf1-4CL5-GW [13], followed by an *Eco*RI restriction site, the Gateway recombination site attB1 and a *Pml*I restriction site. At the 3′-end, it contains a *Pml*I restriction site, followed by the Gateway recombination site attB2, a *Pst*I restriction site and the 15-bp sequence located upstream of the *Pst*I site in pDRf1-4CL5-GW. This sequence was amplified by PCR using the oligonucleotides DsRed-dropout-fw and DsRed-dropout-rv (Additional file 1: Table S2) and inserted by In-Fusion cloning (Clonetech, Mountain View, CA) into a pDRf1-4CL5-GW vector digested with *Eco*RI and *Pst*I to remove the Gateway cassette and yield the pDRf1-4CL5-DsRed vector. BAHD acyltransferase nucleotide sequences were codon-optimized for expression in *S. cerevisiae* using an empirically derived codon usage table. Codon optimization, including restriction site removal, and oligo design (150 mers) were performed using GeneDesign [70]. Oligonucleotides were pooled for synthesis using acoustic deposition (Labcyte Echo 550), and synthesis was performed at the Joint Genome Institute using a two-step polymerase chain assembly (PCA) approach in 2 μL final volume as previously described [71]. PCA products were purified by gel excision and cloned into a *Pml*I-digested pDRf1-4CL5-DsRed vector using Gibson assembly to generate the pDRf1-4CL5-BAHDs vectors. Plating and picking were performed using a QPix 400 system (Molecular Devices, Sunnyvale, CA). Eight colonies per construct were sequence-verified using PACBIO RSII system (Pacific Biosciences, Menlo Park, CA). Synthesized BAHD sequences and protein accession numbers are listed in Additional file 1: Data S1.

For the construct of the pRS423-GW vector, two DNA fragments covering the full backbone including P_*TDH3*_ and T_*CYC1*_ sequences while excluding *XKS1* encoding sequence of the pRS423.XI vector were amplified by PCR using the oligonucleotide pairs pRS423-fw1/pRS423-rv1 and pRS423-fw2/pRS423-rv2 (Additional file 1: Table S2) and the pRS423.XI vector as a template [72]. A third fragment corresponding to the Gateway cassette was amplified using the oligonucleotides GW-fw/GW-rv (Additional file 1: Table S2) and the pDRf1-4CL5-GW vector as a template [13]. The pRS423-GW vector was constructed by assembling the three PCR fragments via In-Fusion cloning (Clonetech, Mountain View, CA). For the construct of the pRS423-FjTAL vector, a nucleotide sequence encoding FjTAL (GenBank accession number AKE50827.1) flanked with the attB1 (5′-end) and attB2 (3′-end) Gateway recombination sites was synthesized for expression in *S. cerevisiae* (Genescript, Piscataway, NJ) and cloned into the Gateway pDONR221 entry vector by BP recombination (Life technologies, Foster City, CA). An entry clone was LR recombined with the pRS423-GW vector to generate the pRS423-FjTAL construct. Plasmids are available upon request through the JBEI-ICE registry (http://public-registry.jbei.org) [73].

### Production of hydroxycinnamate and benzoate conjugates

Overnight cultures from single colonies of recombinant *S. cerevisiae* harboring the pDRf1-4CL5-BAHDs and pDRf1-4CL5 [13] constructs were grown using 2X YNB medium without amino acids, supplemented with 6% glucose and 2X CSM-ura. These cultures were used to inoculated 4 mL of fresh minimal medium at an OD_600_ = 0.15 and shaken at 200 rpm at 30 °C. Precursors were added 5 h post inoculation at the concentrations indicated in Additional file 1: Table S1. These concentrations were selected to be below toxicity levels and avoid growth inhibition. The cultures were shaken at 200 rpm at 30 °C for 24 h in the presence of the precursors for the production of hydrocinnamate and benzoate conjugates. Yeast colonies harboring the pDRf1-4CL5 control vector were grown under similar conditions. Yeast colonies co-transformed with the pRS423-FjTAL plasmid and the pDRf1-4CL5-LaAT1, pDRf1-4CL5-OsHCT4, pDRf1-4CL5-AtSCT, pDRf1-4CL5-HvACT, pDRf1-4CL5-TpHCT2, or pDRf1-4CL5-AtHHT3 vector were grown under similar conditions except that the medium was supplemented with 2X CSM-ura-his. For the detection of metabolites, an aliquot of the culture medium was collected and cleared by centrifugation (21,000×*g* for 5 min at 4 °C), mixed with an equal volume of cold methanol:water (1:1, v/v), and filtered using Amicon Ultra centrifugal filters (3000 Da MW cutoff regenerated cellulose membrane; Millipore, Billerica, MA) prior to analysis using high-performance liquid chromatography (HPLC), electrospray ionization (ESI), and time-of-flight (TOF) mass spectrometry (MS). Alternatively, for the detection of dodecyl hydroxycinnamates, caffeate phenethyl ester and benzoate alcohol esters, the culture medium was cleared by centrifugation and mixed with 2 mL of hexane for extraction with vortexing. The hexane phase was collected after centrifugation and concentrated under a nitrogen stream prior to HPLC-TOF-MS analysis. For the detection of coniferyl ferulate, yeast cells were harvested by centrifugation, washed and resuspended with 1 mL of HPLC grade water, and mixed with 2 mL of hexane for extraction with vortexing. The hexane phase was collected after centrifugation and concentrated under a nitrogen stream prior to HPLC-ESI-TOF-MS analysis.

### LC–MS analysis of hydroxycinnamate and benzoate conjugates

Standard solutions of chlorogenic acid, rosmarinic acid, coniferyl ferulate and cinnamoyl 3β-tropine were prepared in methanol and water (1:1, v/v) and those of caffeate phenethyl ester and benzoate alcohol esters were prepared in hexane. All metabolites were separated via an Agilent 1200 Series Rapid Resolution high performance liquid chromatography (HPLC) system. The systems autosampler tray was set to 6 °C. The HPLC system was coupled to an Agilent Technologies 6210 LC/TOF mass spectrometer (MS) via either electrospray ionization (ESI) or atmospheric pressure chemical ionization (APCI), which was used for MS detection and metabolite identification. Drying gas temperature, drying gas pressure, nebulizing gas pressure and capillary voltage were set to 330 °C, 10 or 11 L/min, 25 lb/in^2^, and 3500 V (in positive or negative ion modes), respectively, unless stated otherwise. For APCI, the vaporizer and corona were set to 350 °C and 4 µA, respectively, unless stated otherwise.

A HPLC-ESI-TOF-MS method previously described in [12] was used for the separation and detection of rosmarinic acid and its derivatives, coumaroyl quinate and malate hydroxycinnamate esters. The same method was used for coniferyl ferulate except that the drying gas temperature was set at 200 °C. A similar method was used for glycerol hydroxycinnamate esters with the following modifications: The mobile phase was composed of water (solvent A) and methanol (solvent B), and metabolites were separated via gradient elution under the following mobile phase compositions: % B was linearly increased from 30% B to 98% B in 12 min, held at 98% B for 0.6 min, decreased from 98% B to 30% B in 0.2 min and held at 30% B for a further 2.8 min. The total run time was 15.6 min. A drying gas temperature of 200 °C was used throughout. A HPLC-ESI-TOF-MS method previously described in [74] was used for the separation and detection of polyamine hydroxycinnamates amides and tropane alkaloids.

Separation of benzoate alcohol esters was conducted on a Phenomenex Kinetex XB-C18 column (100 mm length, 2.1 mm internal diameter, and 2.6 µm particle size; Phenomenex, Torrance, CA). The mobile phase was composed of water (solvent A) and methanol (solvent B). The elution gradient was as follows: % B was linearly increased from 60% B to 100% B in 5.0 min, held at 100% B for 2.0 min, decreased from 100% B to 60% B in 0.2 min and held at 60% B for a further 4.5 min. A flow rate of 0.3 mL/min was used until 7.0 min, increased from 0.3 to 0.45 mL/min in 0.2 min and held at 0.45 mL/min for 4.5 min. The total run time was 11.7 min. The column compartment was set to 55 °C. A sample injection volume of 2 µL was used throughout. APCI was conducted using in the positive ion mode for the detection of [M + H]^+^ ions. The same HPLC column and column compartment temperature were used for the separation of CAPE. The mobile phase was composed of water (solvent A) and acetonitrile (solvent B) and the elution gradient was as follows: % B was increased linearly from 25% B to 100% B in 3.0 min, held at 100% B for 1.0 min, decreased from 100% B to 25% B in 0.2 min and held at 25% B for a further 4.0 min. A flow rate of 0.3 mL/min was used until 4.0 min, increased from 0.3 to 0.45 mL/min in 0.2 min and held at 0.45 mL/min for the remaining 4.0 min. The total run time was 8.2 min. A sample injection volume of 0.5 µL was used throughout. ESI was conducted in the negative ion mode for the detection of [M–H]^−^ ions.

Separation of dodecyl hydroxycinnamates was conducted on a Phenomenex Kinetex XB-C18 column (100 mm length, 3.0 mm internal diameter and 2.6 μm particle size). A sample injection volume of 3 μL was used throughout. The column compartment was set to 55 °C. The mobile phase was composed of water (solvent A) and methanol (solvent B). The elution gradient was as follows: % B was increased linearly from 65% B to 98% B in 1.47 min, held at 98% B for 9.2 min, decreased from 98% B to 65% B in 0.33 min and held at 65% B for a further 2.0 min. A flow rate of 0.42 mL/min was used until 10.67 min, increased from 0.42 to 0.65 mL/min in 0.33 min and held at 0.65 mL/min for 2.0 min. The total run time was 13.0 min. APCI was conducted in the positive ion mode for the detection of [M + H]^+^ ions. The corona and capillary voltage were set to 15 µA and 3000 V, respectively. A nebulizing gas pressure of 30 lb/in^2^ and a drying gas temperature of 200 °C were used throughout.

A HPLC-ESI TOF-MS method was used to detect chlorogenic acid. The chromatography was done using a Phenomenex Kinetex XB-C18 column (100 mm length, 3 mm internal diameter and 2.6 µm particle size) using the aforementioned HPLC system. A sample injection volume of 5 μL was used throughout. The mobile phase was composed of 0.1% formic acid in water (solvent A) and in methanol (solvent B). The elution gradient was as follows: % B was increased linearly from 25 to 77.5% B in 3.0 min, then increased from 77.5 to 97.1% B in 0.3 min, held at 97.1% B for 1.0 min, decreased from 97.1 to 25% B in 0.4 min and held at 25% B for a further 2 min. A flow rate of 0.42 mL/min was used until 4.3 min, then increased from 0.42 to 0.65 mL/min in 0.4 min and held at 0.65 mL/min for 2 min. The total run time was 6.7 min. The column compartment and sample tray were set to 50 °C and 6 °C, respectively. A sample injection volume of 3 µL was used throughout. ESI was conducted in the negative ion mode for the detection of [M–H]^−^ ions. The nebulizing gas pressure was set to 30 lb/in^2^.

Data acquisition and processing were performed by the MassHunter software package (Agilent Technologies Inc., Santa Clara, CA). For each compound detected, the measured masses agreed with the expected theoretical masses within less than 5 ppm mass error.

## Additional file

**Additional file 1: Figure S1.** Synthesis of *p*-coumarate esters and amides from tyrosine in yeast. *S. cerevisiae* strains co-expressing tyrosine ammonia-lyase FjTAL and 4CL5 with LaAT1 **(A)**, OsHCT4 **(B)**, AtSCT **(C)**, HvACT **(D)**, TpHCT2 **(E)** or AtHHT3 **(F)** were fed with 4-hydroxyphenyllactate **(A)**, nothing **(B, C)**, agmatine **(D)**, malate **(E)** or 1-dodecanol **(F)** for the synthesis of *p*-coumaroyl 4’-hydroxyphenyllactate **(A)**, *p*-coumaroyl glycerol **(B)**, *N* ^1^,*N* ^8^-disinapoyl spermidine **(C)**, *p*-coumaroyl agmatine **(D)**, *p*-coumaroyl malate **(E)** and dodecyl *p*-coumarate **(F)**, respectively. **Figure S2.** Synthesis of quinate hydroxycinnamates in yeast. Representative LC-MS chromatograms obtained from analysis of the culture medium of a *S. cerevisiae* strain expressing 4CL5 and NtHQT are shown. The strain was fed with quinate and *p*-coumarate or caffeate for the synthesis of *p*-coumaroyl quinate **(A)** and chlorogenic acid **(B)**, respectively. The LC-MS chromatogram of a solution of authentic chlorogenic acid is also shown **(C)**. **Table S1.** List of acyl acceptors and donors used for the feedings of yeast strains expressing 4CL5 and BAHDs. Concentrations used in the culture medium are indicated. **Table S2.** Oligonucleotides used in this study. **Data S1.** Sequence of the red fluorescent protein dropout cassette and the codon-optimized sequences and accession numbers of BAHD acyltransferases used in this study.

## Abbreviations

AsFMT: *Angelica sinensis* feruloyl-CoA:monolignol transferase
AtHHT3: *Arabidopsis thaliana* caffeoyl-CoA:fatty alcohol transferase
AtSCT: *Arabidopsis thaliana p*-coumaroyl-CoA:spermidine transferase
AtSDT: *Arabidopsis thaliana* sinapoyl-CoA:spermidine transferase
At4CL5: *Arabidopsis thaliana* 4-coumarate:CoA ligase 5
BAHD: BEAT (benzoyl alcohol O-acetyltransferase), AHCT (anthocyanin O-hydroxycinnamoyl transferase), HCBT (anthranilate N-hydroxycinnamoyl/benzoyl transferase), and DAT (deacetyl vindoline 4-O-acetyltransferase)
BPBT: benzoyl-CoA:benzyl alcohol/phenylethanol benzoyltransferase
CAPE: caffeate phenethyl ester
CoA: coenzyme A
EcCS: *Erythroxylum coca* cocaine synthase
FjTAL: *Flavobacterium johnsoniae* tyrosine ammonia-lyase
HCBT: hydroxycinnamoyl/benzoyl-CoA:anthranilate transferase
HvACT: *Hordeum vulgare p*-coumaroyl-CoA:agmatine transferase
LC–MS: liquid chromatography-mass spectrometry
NtHQT: *Nicotiana tabacum* hydroxycinnamoyl-CoA:quinate transferase
OsHCT4: *Oryza sativa* hydroxycinnamoyl-CoA:shikimate transferase 4
RA: rosmarinic acid
TpHCT2: *Trifolium pratense* hydroxycinnamoyl-CoA:shikimate transferase 2

## References

[1] Keasling JD (2010). Manufacturing molecules through metabolic engineering. Science.

[2] Nielsen J, Keasling JD (2016). Engineering cellular metabolism. Cell.

[3] Sun H, Liu Z, Zhao H, Ang EL (2015). Recent advances in combinatorial biosynthesis for drug discovery. Drug Des Devel Ther.

[4] Hong KK, Nielsen J (2012). Metabolic engineering of *Saccharomyces cerevisiae*: a key cell factory platform for future biorefineries. Cell Mol Life Sci.

[5] D’Auria JC (2006). Acyltransferases in plants: a good time to be BAHD. Curr Opin Plant Biol.

[6] Kim BG, Jung WD, Mok H, Ahn JH (2013). Production of hydroxycinnamoyl-shikimates and chlorogenic acid in *Escherichia coli*: production of hydroxycinnamic acid conjugates. Microb Cell Fact.

[7] Cha MN, Kim HJ, Kim BG, Ahn JH (2014). Synthesis of chlorogenic acid and p-coumaroyl shikimates from glucose using engineered *Escherichia coli*. J Microbiol Biotechnol.

[8] Bloch SE, Schmidt-Dannert C (2014). Construction of a chimeric biosynthetic pathway for the de novo biosynthesis of rosmarinic acid in *Escherichia coli*. ChemBioChem.

[9] Jiang J, Bi H, Zhuang Y, Liu S, Liu T, Ma Y (2016). Engineered synthesis of rosmarinic acid in *Escherichia coli* resulting production of a new intermediate, caffeoyl-phenyllactate. Biotechnol Lett.

[10] Zhuang Y, Jiang J, Bi H, Yin H, Liu S, Liu T (2016). Synthesis of rosmarinic acid analogues in *Escherichia coli*. Biotechnol Lett.

[11] Kim IA, Kim BG, Kim M, Ahn JH (2012). Characterization of hydroxycinnamoyltransferase from rice and its application for biological synthesis of hydroxycinnamoyl glycerols. Phytochemistry.

[12] Eudes A, Juminaga D, Baidoo EE, Collins FW, Keasling JD, Loqué D (2013). Production of hydroxycinnamoyl anthranilates from glucose in *Escherichia coli*. Microb Cell Fact.

[13] Eudes A, Baidoo EE, Yang F, Burd H, Hadi MZ, Collins FW, Keasling JD, Loqué D (2011). Production of tranilast [N-(3′,4′-dimethoxycinnamoyl)-anthranilic acid] and its analogs in yeast *Saccharomyces cerevisiae*. Appl Microbiol Biotechnol.

[14] Eudes A, Teixeira Benites V, Wang G, Baidoo EE, Lee TS, Keasling JD, Loqué D (2015). Precursor-directed combinatorial biosynthesis of cinnamoyl, dihydrocinnamoyl, and benzoyl anthranilates in *Saccharomyces cerevisiae*. PLoS ONE.

[15] Eudes A, Pereira JH, Yogiswara S, Wang G, Teixeira Benites V, Baidoo EE, Lee TS, Adams PD, Keasling JD, Loqué D (2016). Exploiting the substrate promiscuity of hydroxycinnamoyl-CoA: shikimate hydroxycinnamoyl transferase to reduce lignin. Plant Cell Physiol.

[16] Moglia A, Goitre L, Gianoglio S, Baldini E, Trapani E, Genre A, Scattina A, Dondo G, Trabalzini L, Beekwilder J, Retta SF (2015). Evaluation of the bioactive properties of avenanthramide analogs produced in recombinant yeast. BioFactors.

[17] El-Seedi HR, El-Said AM, Khalifa SA, Göransson U, Bohlin L, Borg-Karlson AK, Verpoorte R (2012). Biosynthesis, natural sources, dietary intake, pharmacokinetic properties, and biological activities of hydroxycinnamic acids. J Agric Food Chem.

[18] Guzman JD (2014). Natural cinnamic acids, synthetic derivatives and hybrids with antimicrobial activity. Molecules.

[19] Mo H, Tatman D, Jung M, Elson CE (2000). Farnesyl anthranilate suppresses the growth, in vitro and in vivo, of murine B16 melanomas. Cancer Lett.

[20] Singh S (2000). Chemistry, design, and structure-activity relationship of cocaine antagonists. Chem Rev.

[21] Adams TB, Cohen SM, Doull J, Feron VJ, Goodman JI, Marnett LJ, Munro IC, Portoghese PS, Smith RL, Waddell WJ, Wagner BM (2005). Expert panel of the flavor and extract manufacturers association: the FEMA GRAS assessment of benzyl derivatives used as flavor ingredients. Food Chem Toxicol.

[22] Del Olmo A, Calzada J, Nuñez M (2015). Benzoic acid and its derivatives as naturally occurring compounds in foods and as additives: uses, exposure and controversy. Crit Rev Food Sci Nutr.

[23] Jendresen CB, Stahlhut SG, Li M, Gaspar P, Siedler S, Förster J, Maury J, Borodina I, Nielsen AT (2015). Highly active and specific tyrosine ammonia-lyases from diverse origins enable enhanced production of aromatic compounds in bacteria and s*accharomyces cerevisiae*. Appl Environ Microbiol.

[24] Kim GD, Park YS, Jin YH, Park CS (2015). Production and applications of rosmarinic acid and structurally related compounds. Appl Microbiol Biotechnol.

[25] Landmann C, Hücherig S, Fink B, Hoffmann T, Dittlein D, Coiner HA, Schwab W (2011). Substrate promiscuity of a rosmarinic acid synthase from lavender (*Lavandula angustifolia* L.). Planta.

[26] Ma CM, Kawahata T, Hattori M, Otake T, Wang L, Daneshtalab M (2010). Synthesis, anti-HIV and anti-oxidant activities of caffeoyl 5,6-anhydroquinic acid derivatives. Bioorg Med Chem.

[27] Niggeweg R, Michael AJ, Martin C (2004). Engineering plants with increased levels of the antioxidant chlorogenic acid. Nat Biotechnol.

[28] Juminaga D, Baidoo EE, Redding-Johanson AM, Batth TS, Burd H, Mukhopadhyay A, Petzold CJ, Keasling JD (2012). Modular engineering of l-tyrosine production in *Escherichia coli*. Appl Environ Microbiol.

[29] Kikugawa M, Tsutsuki H, Ida T, Nakajima H, Ihara H, Sakamoto T (2016). Water-soluble ferulic acid derivatives improve amyloid-β-induced neuronal cell death and dysmnesia through inhibition of amyloid-β aggregation. Biosci Biotechnol Biochem.

[30] Evans KO, Compton DL, Laszlo JA, Appell M (2016). Feruloyl glycerol and 1,3-diferuloyl glycerol antioxidant behavior in phospholipid vesicles. Chem Phys Lipids.

[31] Ohta S, Fujimaki T, Uy MM, Yanai M, Yukiyoshi A, Hirata T (2007). Antioxidant hydroxycinnamic acid derivatives isolated from Brazilian bee pollen. Nat Prod Res.

[32] Hadjipavlou-Litina D, Garnelis T, Athanassopoulos CM, Papaioannou D (2009). Kukoamine A analogs with lipoxygenase inhibitory activity. J Enzyme Inhib Med Chem.

[33] Yingyongnarongkul BE, Apiratikul N, Aroonrerk N, Suksamrarn A (2008). Synthesis of bis, tris and tetra(dihydrocaffeoyl) polyamine conjugates as antibacterial agents against VRSA. Arch Pharm Res.

[34] Ma CM, Nakamura N, Hattori M (2001). Inhibitory effects on HIV-1 protease of tri-p-coumaroylspermidine from *Artemisia caruifolia* and related amides. Chem Pharm Bull.

[35] Fixon-Owoo S, Levasseur F, Williams K, Sabado TN, Lowe M, Klose M, Joffre Mercier A, Fields P, Atkinson J (2003). Preparation and biological assessment of hydroxycinnamic acid amides of polyamines. Phytochemistry.

[36] Li Z, Fennie MW, Ganem B, Hancock MT, Kobaslija M, Rattendi D, Bacchi CJ, O’Sullivan MC (2001). Polyamines with N-(3-phenylpropyl) substituents are effective competitive inhibitors of trypanothione reductase and trypanocidal agents. Bioorg Med Chem Lett.

[37] Yamamoto A, Nakamura K, Furukawa K, Konishi Y, Ogino T, Higashiura K, Yago H, Okamoto K, Otsuka M (2002). A new nonpeptide tachykinin NK1 receptor antagonist isolated from the plants of *Compositae*. Chem Pharm Bull.

[38] Zhao G, Qin GW, Gai Y, Guo LH (2010). Structural identification of a new tri-p-coumaroylspermidine with serotonin transporter inhibition from safflower. Chem Pharm Bull.

[39] Luo J, Fuell C, Parr A, Hill L, Bailey P, Elliott K, Fairhurst SA, Martin C, Michael AJ (2009). A novel polyamine acyltransferase responsible for the accumulation of spermidine conjugates in Arabidopsis seed. Plant Cell.

[40] Burhenne K, Kristensen BK, Rasmussen SK (2003). A new class of N-hydroxycinnamoyltransferases. Purification, cloning, and expression of a barley agmatine coumaroyltransferase (EC 2.3.1.64). J Biol Chem.

[41] Kim SK, Jin YS, Choi IG, Park YC, Seo JH (2015). Enhanced tolerance of *Saccharomyces cerevisiae* to multiple lignocellulose-derived inhibitors through modulation of spermidine contents. Metab Eng.

[42] Chou SC, Everngam MC, Sturtz G, Beck JJ (2006). Antibacterial activity of components from *Lomatium californicum*. Phytother Res.

[43] Ho CC, Kumaran A, Hwang LS (2009). Bio-assay guided isolation and identification of anti-Alzheimer active compounds from the root of *Angelica sinensis*. Food Chem..

[44] Chen C, Wu C, Lu X, Yan Z, Gao J, Zhao H, Li S (2013). Coniferyl ferulate, a strong inhibitor of glutathione S-transferase isolated from radix *Angelicae sinensis*, reverses multidrug resistance and downregulates P-glycoprotein. Evid Based Complement Alternat Med.

[45] Wilkerson CG, Mansfield SD, Lu F, Withers S, Park JY, Karlen SD, Gonzales-Vigil E, Padmakshan D, Unda F, Rencoret J, Ralph J (2014). Monolignol ferulate transferase introduces chemically labile linkages into the lignin backbone. Science.

[46] Jansen F, Gillessen B, Mueller F, Commandeur U, Fischer R, Kreuzaler F (2014). Metabolic engineering for *p*-coumaryl alcohol production in *Escherichia coli* by introducing an artificial phenylpropanoid pathway. Biotechnol Appl Biochem.

[47] van Summeren-Wesenhagen PV, Voges R, Dennig A, Sokolowsky S, Noack S, Schwaneberg U, Marienhagen J (2015). Combinatorial optimization of synthetic operons for the microbial production of *p*-coumaryl alcohol with *Escherichia coli*. Microb Cell Fact.

[48] Kimura Y, Okuda H, Okuda T, Hatano T, Arichi S (1987). Studies on the activities of tannins and related compounds, X. Effects of caffeetannins and related compounds on arachidonate metabolism in human polymorphonuclear leukocytes. J Nat Prod.

[49] Sullivan M (2009). A novel red clover hydroxycinnamoyl transferase has enzymatic activities consistent with a role in phaselic acid biosynthesis. Plant Physiol.

[50] Liu P, Jarboe LR (2012). Metabolic engineering of biocatalysts for carboxylic acids production. Comput Struct Biotechnol J..

[51] Anselmi C, Centini M, Andreassi M, Buonocore A, La Rosa C, Facino RM, Sega A, Tsuno F (2004). Conformational analysis: a tool for the elucidation of the antioxidant properties of ferulic acid derivatives in membrane models. J Pharm Biomed Anal.

[52] Jayaprakasam B, Vanisree M, Zhang Y, Dewitt DL, Nair MG (2006). Impact of alkyl esters of caffeic and ferulic acids on tumor cell proliferation, cyclooxygenase enzyme, and lipid peroxidation. J Agric Food Chem.

[53] Kosma DK, Molina I, Ohlrogge JB, Pollard M (2012). Identification of an Arabidopsis fatty alcohol:caffeoyl-Coenzyme A acyltransferase required for the synthesis of alkyl hydroxycinnamates in root waxes. Plant Physiol.

[54] Runguphan W, Keasling JD (2014). Metabolic engineering of *Saccharomyces cerevisiae* for production of fatty acid-derived biofuels and chemicals. Metab Eng.

[55] Sheng J, Stevens J, Feng X (2016). Pathway compartmentalization in peroxisome of *Saccharomyces cerevisiae* to produce versatile medium chain fatty alcohols. Sci Rep.

[56] Fodor G, Dharanipragada R (1994). Tropane alkaloids. Nat Prod Rep.

[57] Grynkiewicz G, Gadzikowska M (2008). Tropane alkaloids as medicinally useful natural products and their synthetic derivatives as new drugs. Pharmacol Rep.

[58] Schmidt GW, Jirschitzka J, Porta T, Reichelt M, Luck K, Torre JC, Dolke F, Varesio E, Hopfgartner G, Gershenzon J, D’Auria JC (2015). The last step in cocaine biosynthesis is catalyzed by a BAHD acyltransferase. Plant Physiol.

[59] Qualley AV, Widhalm JR, Adebesin F, Kish CM, Dudareva N (2012). Completion of the core β-oxidative pathway of benzoic acid biosynthesis in plants. Proc Natl Acad Sci USA.

[60] Palazón J, Navarro-Ocaña A, Hernandez-Vazquez L, Mirjalili MH (2008). Application of metabolic engineering to the production of scopolamine. Molecules.

[61] Boatright J, Negre F, Chen X, Kish CM, Wood B, Peel G, Orlova I, Gang D, Rhodes D, Dudareva N (2004). Understanding in vivo benzenoid metabolism in petunia petal tissue. Plant Physiol.

[62] Tolba MF, Azab SS, Khalifa AE, Abdel-Rahman SZ, Abdel-Naim AB (2013). Caffeic acid phenethyl ester, a promising component of propolis with a plethora of biological activities: a review on its anti-inflammatory, neuroprotective, hepatoprotective, and cardioprotective effects. IUBMB Life.

[63] Murtaza G, Karim S, Akram MR, Khan SA, Azhar S, Mumtaz A, Bin Asad MH (2014). Caffeic acid phenethyl ester and therapeutic potentials. Biomed Res Int.

[64] Zhang P, Tang Y, Li NG, Zhu Y, Duan JA (2014). Bioactivity and chemical synthesis of caffeic acid phenethyl ester and its derivatives. Molecules.

[65] Dudareva N, D’Auria JC, Nam KH, Raguso RA, Pichersky E (2002). Acetyl-CoA:benzylalcohol acetyltransferase–an enzyme involved in floral scent production in *Clarkia breweri*. Plant J.

[66] D’Auria JC, Chen F, Pichersky E (2002). Characterization of an acyltransferase capable of synthesizing benzylbenzoate and other volatile esters in flowers and damaged leaves of *Clarkia breweri*. Plant Physiol.

[67] Sun J, Alper HS (2015). Metabolic engineering of strains: from industrial-scale to lab-scale chemical production. J Ind Microbiol Biotechnol.

[68] Kim B, Cho BR, Hahn JS (2014). Metabolic engineering of *Saccharomyces cerevisiae* for the production of 2-phenylethanol via Ehrlich pathway. Biotechnol Bioeng.

[69] Winzeler EA, Shoemaker DD, Astromoff A, Liang H, Anderson K, Andre B, Bangham R, Benito R, Boeke JD, Bussey H, Chu AM, Connelly C, Davis K, Dietrich F, Dow SW, El Bakkoury M, Foury F, Friend SH, Gentalen E, Giaever G, Hegemann JH, Jones T, Laub M, Liao H, Liebundguth N, Lockhart DJ, Lucau-Danila A, Lussier M, M’Rabet N, Menard P, Mittmann M, Pai C, Rebischung C, Revuelta JL, Riles L, Roberts CJ, Ross-MacDonald P, Scherens B, Snyder M, Sookhai-Mahadeo S, Storms RK, Véronneau S, Voet M, Volckaert G, Ward TR, Wysocki R, Yen GS, Yu K, Zimmermann K, Philippsen P, Johnston M, Davis RW (1999). Functionnal characterization of the *S. cerevisiae* genome by deletion and parallel analysis. Science.

[70] Richardson SM, Nunley PW, Yarrington RM, Boeke JD, Bader JS (2010). GeneDesign 3.0 is an updated synthetic biology toolkit. Nucleic Acids Res.

[71] Heins RA, Cheng X, Nath S, Deng K, Bowen BP, Chivian DC, Datta S, Friedland GD, D’Haeseleer P, Wu D, Tran-Gyamfi M, Scullin CS, Singh S, Shi W, Hamilton MG, Bendall ML, Sczyrba A, Thompson J, Feldman T, Guenther JM, Gladden JM, Cheng JF, Adams PD, Rubin EM, Simmons BA, Sale KL, Northen TR, Deutsch S (2014). Phylogenomically guided identification of industrially relevant GH1 β-glucosidases through DNA synthesis and nanostructure-initiator mass spectrometry. ACS Chem Biol.

[72] Reider Apel A, Ouellet M, Szmidt-Middleton H, Keasling JD, Mukhopadhyay A (2016). Evolved hexose transporter enhances xylose uptake and glucose/xylose co-utilization in *Saccharomyces cerevisiae*. Sci Rep.

[73] Ham TS, Dmytriv Z, Plahar H, Chen J, Hillson NJ, Keasling JD (2012). Design, implementation and practice of JBEI-ICE: an open source biological part registry platform and tools. Nucleic Acids Res.

[74] Bokinsky G, Baidoo EE, Akella S, Burd H, Weaver D, Alonso-Gutierrez J, Garcia-Martin H, Lee TS, Keasling JD (2003). HipA-triggered growth arrest and b-lactam tolerance in *Escherichia coli* are mediated by RelA-dependent ppGpp synthesis. J Bacteriol.

