# Supplementary material for: Exploiting members of the BAHD acyltransferase family to synthesize multiple hydroxycinnamate and benzoate conjugates in yeast
Source: Microb Cell Fact. 2016 Nov 21;15:198. doi: 10.1186/s12934-016-0593-5 (PMC5117604; doi:10.1186/s12934-016-0593-5)
Supplement: Supplementary file 1 — Additional file 1: Figure S1. Synthesis of p-coumarate esters and amides from tyrosine in yeast. S. cerevisiae strains co-expressing tyrosine ammonia-lyase FjTAL and 4CL5 with LaAT1 (A), OsHCT4 (B), AtSCT (C), HvACT (D), TpHCT2 (E) or AtHHT3 (F) were fed with 4-hydroxyphenyllactate (A), nothing (B, C), agmatine (D), malate (E) or 1-dodecanol (F) for the synthesis of p-coumaroyl 4’-hydroxyphenyllactate (A), p-coumaroyl glycerol (B), N 1,N 8-disinapoyl spermidine (C), p-coumaroyl agmatine (D), p-coumaroyl malate (E) and dodecyl p-coumarate (F), respectively. Figure S2. Synthesis of quinate hydroxycinnamates in yeast. Representative LC-MS chromatograms obtained from analysis of the culture medium of a S. cerevisiae strain expressing 4CL5 and NtHQT are shown. The strain was fed with quinate and p-coumarate or caffeate for the synthesis of p-coumaroyl quinate (A) and chlorogenic acid (B), respectively. The LC-MS chromatogram of a solution of authentic chlorogenic acid is also shown (C). Table S1. List of acyl acceptors and donors used for the feedings of yeast strains expressing 4CL5 and BAHDs. Concentrations used in the culture medium are indicated. Table S2. Oligonucleotides used in this study. Data S1. Sequence of the red fluorescent protein dropout cassette and the codon-optimized sequences and accession numbers of BAHD acyltransferases used in this study. [file 12934_2016_593_MOESM1_ESM.pdf]

Data S1: Sequence of the red fluorescent protein dropout cassette and the codon-optimized sequences and accession numbers of BAHD acyltransferases used in this study.

>RFP fluorescent dropout cassette (*Pml*I-DsRed-droupout):

CCCCAGCCTCGAACTAGTGAATTCGCGGCCATCACAAGTTTGTACAAAAAAGCAGGCTTCcacg
tgGATCTTCCCTATCAGTGATAGAGATTGACATCCCTATCAGTGATAGAGATACTGAGCACGGA
TCTGAAAGAGGAGAAAGGATCTATGGCGAGTAGCGAAGACGTTATCAAAGAGTTCATGCGTTTC
AAAGTTCGTATGGAAGGTTCCGTTAACGGTCACGAGTTCGAAATCGAAGGTGAAGGTGAAGGTC
GTCCGTACGAAGGTACCCAGACCGCTAAACTGAAAGTTACCAAAGGTGGTCCGCTGCCGTTCGC
TTGGGACATCCTGTCCCCGCAGTTCCAGTACGGTTCCAAAGCTTACGTTAAACACCCGGCTGAC
ATCCCGGACTACCTGAAACTGTCCTTCCCGGAAGGTTTCAAATGGGAACGTGTTATGAACTTCG
AAGACGGTGGTGTTGTTACCGTTACCCAGGACTCCTCCCTGCAAGACGGTGAGTTCATCTACAA
AGTTAAACTGCGTGGTACCAACTTCCCGTCCGACGGTCCGGTTATGCAGAAAAAAACCATGGGT
TGGGAAGCTTCCACCGAACGTATGTACCCGGAAGACGGTGCTCTGAAAGGTGAAATCAAAATGC
GTCTGAAACTGAAAGACGGTGGTCACTACGACGCTGAAGTTAAAACCACCTACATGGCTAAAAA
ACCGGTTCAGCTGCCGGGTGCTTACAAAACCGACATCAAACTGGACATCACCTCCCACAACGAA
GACTACACCATCGTTGAACAGTACGAACGTGCTGAAGGTCGTCACTCCACCGGTGCTTAATAAG
GATCTCCAGGCATCAAATAAAACGAAAGGCTCAGTCGAAAGACTGGGCCTTTCGTTTTATCTGT
TGTTTGTCGGTGAACGCTCTCTACTAGAGTCACACTGGCTCACCTTCGGGTGGGCCTTTCTGCG
TTTATAGcacgtggACCCAGCTTTCTTGTACAAAGTGGTGTAGTTAATTCATGATGGCCGCTGC
AGGTCGACCTCGAGGGG

>AtSDT (NCBI Reference Sequence: NP_179932.1):

atgccaatccacattggatcttcaatcccattgatggttgaaaaaatgttgaccgagatggtta
agccttcaaaacatatccctcagcaaacccttaatttatctacattagataacgacccttataa
cgaggtcatttataaggcttgctacgtgtttaaggcaaaaaatgtcgctgatgacgataataga
cctgaagccttgttaagagaggcgttgtccgacttattaggttattattatcctttatccggtt
ctttgaaaagacaagaatctgataggaagttacaattatcttgtggtggtgatggaggtggtgt
tcctttcacagttgccactgctaacgtcgaattgagttctttgaaaaacttagaaaacatcgat
tccgacacagccttgaatttttaccagtcttgcacgttgatatcgacggttaccgtcctttcg
cattacaggtcaccaagtttgagtgtggtggtttcattttgggtatggccatgtctcatgcaat
gtgtgatggttatggtgagggacatatcatgtgtgcattgacagatttggctggtggtaaaaag
aagcctatggtgactcctatttgggagagagaaaggttggtcggaaaacctgaagacgatcaac
ctcctttcgttcctggtgacgataccgccgcaagtccatatttaccaacagacgattgggttac
tgaaaagattaccatcagggccgactctattagaagattgaaggaggccaccttgaaagagtac
gacttttctaatgagacaatcactactttcgaggtcatcggtgcttatttatggaagtccaggg
tcaaagcattgaatttagatagagacggtgtgactgttttgggtttatcagtcggtatccgtaa
cgtcgtggatcctccacttcctgacggttattatggtaacgcctacattgatatgtatgttcct
ttaactgctagagaagttgaggaattcaccattagtgacatcgtgaagttgattaaagaggcca
agaggaacgctcacgataaggattatttgcaagaagaattagccaatacagaaaaaatcatcaa
gatgaatttaactattaagggtaagaaagacggtttgttctgtttaaccgattggagaaacatc
ggtattttcggatcaatggacttcggttgggatgagccagttaatatcgttcctgtggttccat
ctgagactgcaaggacagttaacatgttcatgaggccatctagattggaatctgatatggtcgg

aggtgttcagattgtcgttactttgccaaggatcgcaatggtcaagttcaaagaggaaatggag
gccttggaataa

>AtSCT (NCBI Reference Sequence: NP_180087.1):
atggctaatcaaaggaaaccaatcttgcctttattgcttgaaaagaaacctgttgaattagtca
agccttctaaacatacccattgtgaaactttgtcattatctaccttggataacgaccctttcaa
tgaggttatgtacgctactatctacgtttttaaggccaatggaaagaatttagatgatccagtg
agtttgttacgtaaggcattgagtgagcttttagtgcattactaccctttgtcaggtaaattga
tgcgttctgaatctaacggtaagttacaattggtctatttaggtgagggagtgccattcgaagt
cgccacctccactttggacttatcttcattaaattacattgagaatttggatgatcaagttgcc
ttaaggttagttcctgagattgaaattgattatgagtctaatgtctgctaccacccattggcct
tacaagttactaagtttgcctgtggtggatttactatcggtaccgctttaacccatgcagtctg
tgatggttatggtgttgctcaaatcatccacgccttgaccgagttagcagctggtaagacagag
ccttccgtcaagagtgtctggcaaagagaaagattagttggtaagattgataataaacctggta
aggtccctggttcccacatcgacggtttcttggctactagtgcatatttgcctaccaccgatgt
tgtcactgaaaccattaatatcagagctggtgacattaaacgtttaaaagattcaatgatgaag
gagtgtgaatacttaaaggagtcattcaccacttacgaagttttgtcttcttatatctggaaat
taagaagtcgtgctttaaagttaaatcctgatggtattaccgtcttgggtgtcgccgtgggtat
tagacacgttttagatccacctttaccaaaaggttattatggaaacgcttacatcgatgtctac
gttgaattgaccgttagagagttggaagagtcatctatttcaaatatcgccaacagagttaaga
aagccaaaaaaacagcctatgaaaaaggttatatcgaagaggagttgaagaatactgaaaggtt
gatgagagatgactcaatgtttgagggtgtttctgatggtttgttctttttgaccgactggcgt
aacatcggttggttcggttctatggatttcggatggaacgagcctgtgaatttaagaccattga
cacaacgtgaatccactgtccatgttggtatgatcttgaaaccatcaaaatctgatccatctat
ggaaggtggtgtcaaggttattatgaagttgcctagagatgccatggtggaattcaaaaggaa
atggcaactatgaagaaattatacttcggagataccaactaa

>AtHHT3 (NCBI Reference Sequence: NP_201161.1):
atggctgattcatttgaattaatcgtgactagaaaagagccagttttggtgtctccagcatccg
aaaccccaaagggattacattatttgagtaatttagaccaaaacattgctatcattgtgaaaac
attttattatttcaagtctaactctagatccaatgaggagtcatacgaggttatcaagaagtcc
ttgtctgaggttttagtccactattatcctgccgctggtaggttaaccatttccccagagggta
agatcgccgtcgattgtactggtgagggtgttgttgttgttgaggcagaggctaactgtggtat
cgaaaagattaagaaggccatttcagagattgatcaacctgaaaccttggagaaattggtctat
gacgttccaggtgctcgtaatatcttggaaattcctccagttgtggtgcaagtgactaacttca
agtgtggtggatttgttttaggtttaggtatgaatcacaatatgttcgatggtattgcagccat
ggaatttttgaattcctgggccgaaactgcccgtggtttacctttgtctgtgccaccttttta
gatcgtactttgcttagacctcgtactccacctaagatcgagttcccacataacgagtttgaag
atttagaggatatttccggtactggtaagttgtactccgatgaaaaattagtttacaaatcttt
cttgttcggtcctgagaagttggagagattaaagatcatggccgagacaaggtctaccaccttt
cagactttgactggtttcttgtggagggctcgttgtcaagccttaggtttaaaaccagatcaaa

gaatcaagttgttattcgccgcagacggtagatctagattcgtccctgaattgcctaagggtta
ttcaggtaatggaatcgtgttcacctactgcgtcaccactgctggtgaggtgactttaaatcca
ttatctcactccgtctgtttggttaagcgtgctgttgaaatggtcaacgacggttttatgagat
cagcgatcgactatttcgaggtgacccgtgccagaccatctttaaccgctactttattgattac
atcatgggccaagttatctttccatactaaggacttcggttggggagaacctgtcgtttcaggt
cctgtgggtttgcctgagaaagaagttatcttgttcttgccatgcggatccgatacaaagagta
tcaacgtgttattaggtttacctggtagtgctatgaaggtttttcaaggtatcatggatatcta
a

>HvACT (GenBank: AAO73071.1)
atgaaaatcaccgttcattcatctaaggctgttaagcctgagtacggtgcttgcggtttagctc
ctggttgtactgccgatgttgttccattgacagtcttggacaaagctaattttgatacttatat
ttctgttatttacgccttccatgcacctgctccacctaacgctgtcttagaagccggattaggt
agggctttggttgattatcgtgaatgggctggtaggttgggtgtggatgcttccggtggtcgtg
caatcttattgaatgatgctggagcacgtttcgtcgaggcaaccgctgatgtggccttggatag
tgttatgcctttaaagcctacttctgaagttttatcattacacccatctggtgacgatggacct
gaggagttgatgttgattcaggtgaccagatttgcatgtggttctttggttgtcggttttacca
ctcaacacatcgtcagtgacggtcgtagtaccggtaatttcttcgtcgcttggtcccaagccac
tagaggtgccgctattgatccagttccagtccacgatcgtgcatctttcttccaccctagagaa
cctttacatgtggaatacgaacatagaggtgtcgaattcaagccatatgagaaagctcacgatg
tcgtctgtggtgcagatggtgatgaagatgaggtggtcgtgaacaaagtccatttctctcgtga
gtttatttctaaattaaaggctcaagcctctgcgggagctcctagaccatgttctactttgcaa
tgtgttgtggcacacttgtggaggtctatgaccatggctagaggtttagatggtggtgagacaa
cctctgtcgctattgccgttgatggaagggccagaatgtctcctcaagttccagatggttacac
tggtaacgtcatcttatgggctagacctaccaccactgccggtgaattggttacccgtcctgtc
aagcatgcagttgagttaatctctagagaggttgctcgtattaatgatggatatttcaagtcat
tcatcgatttcgccaactctggtgctgttgagaaggagagattggtcgccactgccgacgctgc
agatatggtcttatccccaaatatcgaagtcgattcttggttgttaatccctttctacgacatg
gatttcggaggtggtaggccattcttctttatgccatcttatttacctgtcgagggtttattga
ttttattaccatccttcttgggtgatggttcagtggatgcttacgtgcctttattctctagaga
catgaatacctttaagaattgttgctattctttagattaa

>OsHCT4 (NCBI Reference Sequence: XP_015641749.1)
atggccactgtcgatgtgttgacctccgaggtcgttgtcccagctggtgaaactccagccggag
ccgtgtggttaagtaacttagacttggccgctagaaggggttacaccccaacagttttttttta
ccgtcataatggtgaacctggtttctttgctgccgatgctatgagagattccttggctagggct
ttggtcgccttctaccctgtggctggaagattaggtttggatggtgatggtagagttcaggtcg
attgtactggtgaaggtgtggtcttcgccactgcacgttctggtcactatgccttagatgactt
gatgggtgagtttgtcccttgtgacgagatgagagatttattcgtgccagctgcccctgcagct
gcttcatgctgtccaagaggaggtgccttgttgttagttcaggtcacttacttaagatgtggtg
gtgttgttttgggtatggctttacatcactcaatcgccgatggaaggtctgcagctcatttcgt

ggaaacttgggcatctatcgctaggggtgccccagcagctgacgcacctgtgccaccttgtttt
gatcatagattgttagccgcaagaccagctcgtgcagttttatacgatcaccctgaatataagc
ctgaaccagctcctcctgcaagggctgcaactgccagtacctatgcttcagcaatcatcacatt
aaccaaacaacaagtgggtgccttacgtgctgcctgtgctggtgcctctaccttcagagcagtc
gttgctttagtgtggcagtgtgcctgtagggcccgtgccttgcctccagaagccgagacacgtt
tgcattcaatgatcgacaccagacagagattaagtccacctttgccacctggatattttggtaa
tgccgtgatcagaacttcaaccgccgcaactgccggtgaggttgtgagtagtcctgttggtcat
gccgccagaagggctagagctgccacctcccaaggagaagattatgctagatctgtcgttgatt
acttagagggtgtggatgccatgaatttgccaaggtctggtgtttcaagagccgatttaagagc
tatctcttggttaggtatgtctttagctgatgccgatttcggatggggttcacctgcttttatg
ggtccagctattatgtactattctggtttcgtgtatgttatgaatgcgcctggaaaggacggtg
cagtcgcattggccttatctttggaaccagaatcaatgccagaatttagaaaggtgttcgctga
tgaagttgctagacttgcttaa

>AsFMT (GenBank: AHL24755.1)
atgactatcatggaggttcaggtcgtttctaaaaagatggtgaagccatcagttcctacccctg
atcatcacaaaacatgtaagttgaccgccttcgaccaaattgccccacctgatcaagtgccaat
catctacttctataattcttctaacatccataatattagagagcaattggtcaagagtttgtca
gagacattaactaaattttatccattggcaggtaggttcgttcaggacggttttatgtcgact
gtaacgacgaaggtgtcttgtacgtggaggctgaggtcaatatccctttaaacgaattcatcgg
tcaagccaaaaagaacattcagttgatcaatgatttggttccaaaaaagaacttcaaggacatc
cattcttatgaaaatccaatcgtcggattacaaatgtcttattttaaatgcggtggattagcta
tctgtatgtacttgtctcatgtggttgcagatggttacaccgctgccgccttcacaaaggagtg
gtccaataccactaatggtattatcaatggcgatcaattggtcagttcatcccctattaacttt
gagttggctaccttggtccctgcacgtgacttaagtaccgttatcaagcctgctgttatgccac
cttccaaaatcaaggagacaaaggtggtcacaagaaggttcttgtttgatgagaatgcaatctc
tgctttcaaggaccacgtgatcaaatccgaatctgtcaacagaccaacaagagttgaggttgtg
acttccgtcttgtggaaggcccttatcaatcaatctaagttgccttcttccacactttatttc
acttgaatttcagaggtaagaccggaattaatacccctcctttagataatcacttctctttatg
tggtaatttctacacacaagttccaactcgtttcagaggtggaaatcaaactaagcaagacttg
gaattgcatgagttagtcaaattgttacgtggtaagttgaggaacacattgaaaaattgtagtg
aaattaataccgccgatggtttattcttggaggctgcctctaacttcaatattatccaagaaga
cttggaggatgagcaagtggacgttcgtatcttcaccacattgtgtcgtatgccattatacgag
actgagtttggttggggtaagcctgaatgggtcactatcccagagatgcacttagaaatcgttt
tcttattggacactaaatgcggtaccggtatcgaggctttggtatctatggatgaagcagacat
gttgcagtttgagttggatccaactatttctgcattcgcttcttaa

>EcCS (GenBank: AGT56097.1)
atggagatgtcaaaaaaaaagcttgaaattatcttaagaaaaaccattaagccttcttccagta
ctcctcagcacttgcaaacctttgagttatctttctgggatgagcctttgcctcctgactacgg
taccatcatttcttctatcagacaaatggttcaaaaaacgacgatgacgaggctttgtctatc

ttctttcagagatcaagttcattacaaaattctttgtccaagactttaatccactattatccat
tggcgggtagattgaaggatgacggtacagctgtcgattgcaatgatgaaggtgcatacttcgt
tgaggctcgtatcgattgtcagttatctactttgttgaatcatcctgatgccgacttcttgtct
cactatttctgtcctgctttagattcaaataacttgccttctggttgtatgttagcgattcaat
taaccttatttaattgtggtggtatcgccatctctgtcagtccttctcacaagatcgccgacgc
ttcttccgcttgcactttcgtccagtcatgggcttcaatgaccactattggtgaggtggctcct
aaaccaatctttttggaaccttcctcttctcctcctcgttctttaagaaacgccccaatgttaa
ccgagattcctggtgagttcgtgaagcgtagatttgttttcccagcttctaagattgcacaatt
gcgtactaagaccacaggtccttcttctcctaccggtaagcaacatatgtcagacgccgatttg
gtcatggcattattcatgaagtgtgctatcttggcttctagatctttgtctaaatcatcctctg
gtccatacgtgttgttccaagttgttgatttacgtaaaagagtcagacctccattgcctgctaa
tacaatcggtaacgtggttttgtactacacccacccagattgaggaaaaccaaattgagttgaat
gagttggcaggtaagttccgtaagtcattaaacgaattctgtaacttagctgccaattcaagtt
taaatgaggaaccagaattcatcattcaaggatctccttattgttgtaccaacttgtgcggttt
ccctttctacgagatcgacttcggatggggtaagccttcttgggtgactaccgagttattgtgg
tttagaaatattatcgttttgcagaagactaaagatggtgatggaatcgagttgtgggtttcca
tggatgagaaggagatggcattattcgaacaagatcatgacatcattgcctatgcttctaacaa
tccaagtgttttagccgcctacagtagaatgtaa

>TpHCT2 (GenBank: ACI16631.1)
atggttaccatcaagaactcatataccgttattccagaagaacctaccccacaaggaagattgt
ggctttctgataaagatcaggtggccactcaacaccatactccaaccatctacatttataagcc
taatcaaaaccaagaaaacgttatcgaaacattgaagaattctttgagtaagatcttagtccac
tattatccaatcgctggtagattgtgctactctgatgaagaagttgactcatgtagggtggaat
taaatttgaatgcaaagggtgctatttattggaggcagagactactaaaaccatccacgacta
cggagattttcaccttccgacttaaccaaggaattgatccctatcattgattataatcagcca
ttccaagaaatgcctttgttggtcgtgcagttaactagttttaaaaataaccaaggttttgctt
tgggagttgctttctcacactctttatcagacggtaccggtgccgttaaattcatcaatagttg
ggccaagattgccaggggtgaaacattagaaccaaacgaattaccttttttggacagaactttg
attaaattttctcatacaccatcaaaggtccctaggttcgaacacatcgaattaaagcctttgc
ctttaattatcggtcgtaaagatacttctgaggaaagaaaaaaaaagactaccgcgacattgtt
aaagttgtcatatgaccaggtcgagaaattaaagaagaaggccaatgatttcttaagtatgaaa
aagaaaggttcacgtcctttttctaagttcgaggcaatcggtgcccacttgtggagatgtgcct
ctaaagccagaggtttagaagatgaccaagagagtgtggttaggttccatgccgatatcagaag
gagaatcaatccacctttgccacaaaactttttcgctaatgcattggctttaaccgccactaag
ggttgtgtcggtgaaatcacctctaagcctttgggttacgtggctcagaagatcagagaaggta
ctgaattggtcaaagatgatttcatcaaatcacaaattgatgtgatcagaagtttcagaaagat
ggatgacgccatgaagttgttccttggtgatgagactgaaaaggctccatatttcggtaatcca
aacttccaggttgcttcctggaccggaatgccattttacgaggccgatttcggttacggtaaac
ctatttattttggttacgctggtgtgtctccacatgaccgtgcctatatcactttatctcctga

cggtgatggatcagtcatcgtttctttgcacttccaaatggctcacttagagttgtttaaaaag
tatttttatgaagatatttaa

>LaAT1 (GenBank: ABI48360.1)

atgaagatcgagattaaagagtctactatggttagacctgctgctgaaacccccttctggtagtt
tgtggctttcaaacttagacttattgtctcctgccaattaccataccttgtctgtccacttcta
ttcccacgatggttctgcaaacttcttcgacgcgaccgctttaaaagaggccttatccagagcc
ttggtggatttctacccatacgccggtaggttaaagttgaacaaggagaataggttagagatcg
agtgcaacggtgagggtattcttttggttgaagctgagtgttcaggtgctttggatgaattagg
tgatttcactccaagacctgagttgaatttgatccctaaggttgattattctaaaggtatgtcc
acctatcctttgatgttattccagattactagattcaagtgtggtggagtcgcattaggtgtcg
caaacgagcaccacttatctgacggtgttgctgctttgcattttatcaatacttgggcccatta
ctctagaggtgtgccagctccttccccacctccacactttgacagaaccgctttgagtgctcgt
aaccctcctcaacctcaattctcacatgccgaatatcaacctcctcctactttggaaaacccat
tgcctgccaccgatattgcccatagtaaatttaagttgacaagagctcaattgaactcccttaa
ggctaagtgtgccgctggagactcagacggtcacactaacggtactgccaacggtaagagtgat
gctaatggtaccgcagacggaaagtccgatgctaatggtactgccaatggtaagtctgcagcta
agagatactcaaccttcgaagtcttagctggacatatttggaggtcagtttgtacagctagagg
tttgccagcagaacaggaaactaaattgcacatccctttcgacggtagatctcgtttgaacttg
cctcctggttattttggaaatgctattttcttcgccacacctattgctacctgtggtgaaattg
agtcaaattctctttcatatgcagttagaagggtcggagatggtattgcacgtttagacgaaga
gtatttgaagtcttctttagatttcttagaattacaaccagacatctccaaattggctcagggt
gcacacagttttcgttgtcctaacttgtgggttatttcatgggtttggttaccaatctatgaac
ctgacttcggttggggtaaggccgtccatatgggtccatgggctgctccattcgagggtaagtc
ttatttacttccaaatcctgagaacgacggttctttgttcgtgtcaatcactttgcacaaacaa
cacatggaaaggtttcaaaagttattctacgagatttaa

>NtHQT (GenBank: CAE46932.1)

atgggttctgagaagatgatgaaaattaatatcaaagagtccaccttagtgaagccatctaaac
ctaccccaaccaagagattgtggtcttcaaatttagatttgatcgttggtagaatccacttgtt
aaccgtgtacttttataaaccaaacggttcttctaatttttcgactcaaagattatgaaggaa
gctttgtctaatgttttggttagtttttacccaatggccggtagattagctagggatgaacaag
gaagaattgagatcaactgtaatggtgaaggtgtcttattcgtggaggccgagtctgacgcttt
cgttgatgacttcggtgacttcacccccttcattagaattgcgtaagttgatcccaaccgtcgat
acttctggtgatatctctacatttccattgatcatctttcaagtcaccagattcaagtgcggag
gtgtgtctttgggtggtggtgttttccacacattgtccgatggtttgtcttctatccatttcat
caatacatggagtgatatcgccagaggtttatctgtcgccattcctccatttatcgataggacc
ttgttaagagccagagatccacctaccagttctttcgaacacgtggagtatcacccacctccat
ctcttatttcctcttctaaatctttggagtctacttcaccaaaaccttctacaactacaatgtt
gaagttttcttctgatcagttgggtttgttgaagtcaaaatcaaaacacgatggatcaacttac
gaaatcttagccgcccacatctggcgttgtacctgcaaggctcgtgcattgtccgatgaccaat

tgacaaaattacacgttgccaccgatggtagatcaagattgtgtccacctttgcctcctggata
tttaggtaacgtcgttttcactggtactcctatggcaaagtcaagtgagttgttgcaggaacca
ttaaccaattctgcgaaacgtatccattcagctttgtcaaagatggatgataactacttaagat
ctgcattggattatttagagttgttacctgacttgtctgctttaattaggggtccaacttactt
cgcatccccaaatttaaatatcaattcttggacaagattacctgtgcatgattctgactttggt
tggggtcgtccaattcacatgggtccagcttgtatcttatatgaaggtactgtctacatcttac
ctagtccaaattctaaagaccgtaacttgaggttggccgtgtgtttagatgcagatcatatgcc
tttattcgaaaagtatttatacgagttctaa

>BPBT (GenBank: AAT68601.1)
atggacagtaagcaatcatctgaattggtcttcaccgtgagaaggcaggagccagagttgatcg
cccctgcaaagcctacaccaagagaaactaaattttatccgatatcgatgatcaagaaggttt
gcgttttcagattcctgttatcaatttctacagaaaggactcttctatgggaggtaaagatcca
gtggaggtgatcaagaaagcaatcgctgaaactttggtttttattaccctttcgccggtagat
taagagaaggaaatgatagaaagttgatggttgactgtaccggtgagggtgtgatgttcgttga
agctaacgctgatgtgactttggaggaatttggtgatgagttacaaccaccttttccttgcttg
gaggaattattgtatgatgttcctggttctgctggtgtcttgcattgccctttattgttaatcc
aagttaccagattgaggtgtggtggtttcatcttcgccttgcgtttaaatcacactatgtctga
tgcaccaggtttggtgcagtttatgaccgctgtcggagagatggcccgtggtgcaactgctcct
tcaacattgccagtttggtgtagagaattattgaatgctaggaatcctccacaagtgacttgta
cccatcatgagtatgaagaggtccctgatactaagggtaccttaattcctttagacgatatggt
tcaccgttctttttttcttcggaccaactgaagtctctgcattacgtagatttgtgccacctcat
ttgcacaactgttctactttcgaggttttaaccgctgccttgtggagatgtagaaccatctcta
tcaagccagatcctgaagaggaggttcgtgttttgtgtattgttaacgctagatcacgtttcaa
tccacaattaccttctggttactatggtaacgcattcgctttccctgttgccgtcaccacagct
gaaaaattatgtaagaatccattgggttacgcattggagttagttaaaaagaccaagtctgatg
tcactgaagaatatatgaaatctgttgccgacttgatggttattaagggtagacctcattttac
agtcgttaggacctatttggtttcagatgtcacaagagctggtttcggagaggttgatttcggt
tggggtaaagctgtctatggtggacctgccaagggtggtgtcggtgccatccctggtgtggctt
cattctatatcccattccgtaacaagaagggtgaaaatggtatcgttgtccctatctgccttcc
tggtttcgcaatggagaagttcgtgaaggagttagattctatgttaaagggagacgctcaatta
gacaacaagaagtatgccttcattactccagctttgtaa

Table S1: List of acyl acceptors and donors used for the feedings of yeast strains expressing 4CL5 and BAHDs. Concentrations used in the culture medium are indicated.

**Acyl Acceptors**	**Concentrations used for feedings (mM)**	**Suppliers**
Spermine	1	Sigma-Aldrich, St. Louis, MO
Putrescine	1	Sigma-Aldrich, St. Louis, MO
Spermidine	1	Sigma-Aldrich, St. Louis, MO
Agmatine	1	Sigma-Aldrich, St. Louis, MO
Glycerol	34.2	Sigma-Aldrich, St. Louis, MO
Coniferyl alcohol	0.5	Alfa Aesar, Haverhill, MA
3β-tropine	0.5	Chem-Impex International, Wood Dale, IL
Malate	1	Sigma-Aldrich, St. Louis, MO
4-Hydroxyphenyllactate	1	Sigma-Aldrich, St. Louis, MO
3,4-Dihydroxyphenyllactate (Danshensu sodium salt)	1	Sigma-Aldrich, St. Louis, MO
Quinate	1	Sigma-Aldrich, St. Louis, MO
1-Dodecanol	0.56	Sigma-Aldrich, St. Louis, MO
Butanol	13.6	Sigma-Aldrich, St. Louis, MO
Isopentanol	11.3	Sigma-Aldrich, St. Louis, MO
Ethanol	42.8	Sigma-Aldrich, St. Louis, MO
2-Phenylethanol	10.4	TCI America, Portland, OR
**Acyl Donors**		
p-Coumarate	1	TCI America, Portland, OR
Caffeate	2	MP Biomedicals, Burlingame, CA
Ferulate	1	TCI America, Portland, OR
Cinnamate	0.2	Sigma-Aldrich, St. Louis, MO
Benzoic acid	0.2	Sigma-Aldrich, St. Louis, MO

Table S2: Oligonucleotides used in this study.

**Oligonucleotide name**	**Sequence (5’-3’)**
DsRed-dropout-fw	CCCCAGCCTCGAACTAGTGA
DsRed-dropout-rv	CCCCTCGAGGTCGACCTGCA
pRS423-fw1	GAATTCGCTAGAGTCATCATGTAATTAGTTATG
pRS423-rv1	GGTGCACGAGTGGGTTACATC
pRS423-fw2	AACCCACTCGTGCACCCAAC
pRS423-rv2	GGATCCGCTAGCTCGAAACTAAGTTCTGG
GW-fw	cgagctagcGGATCCCACAAGTTTGTACAAAAAAGCAGGCTTC
GW-rv	gactctagcGAATTCTCAATTAACTACACCACTTTGTACAAGAAAGCTGGGTC

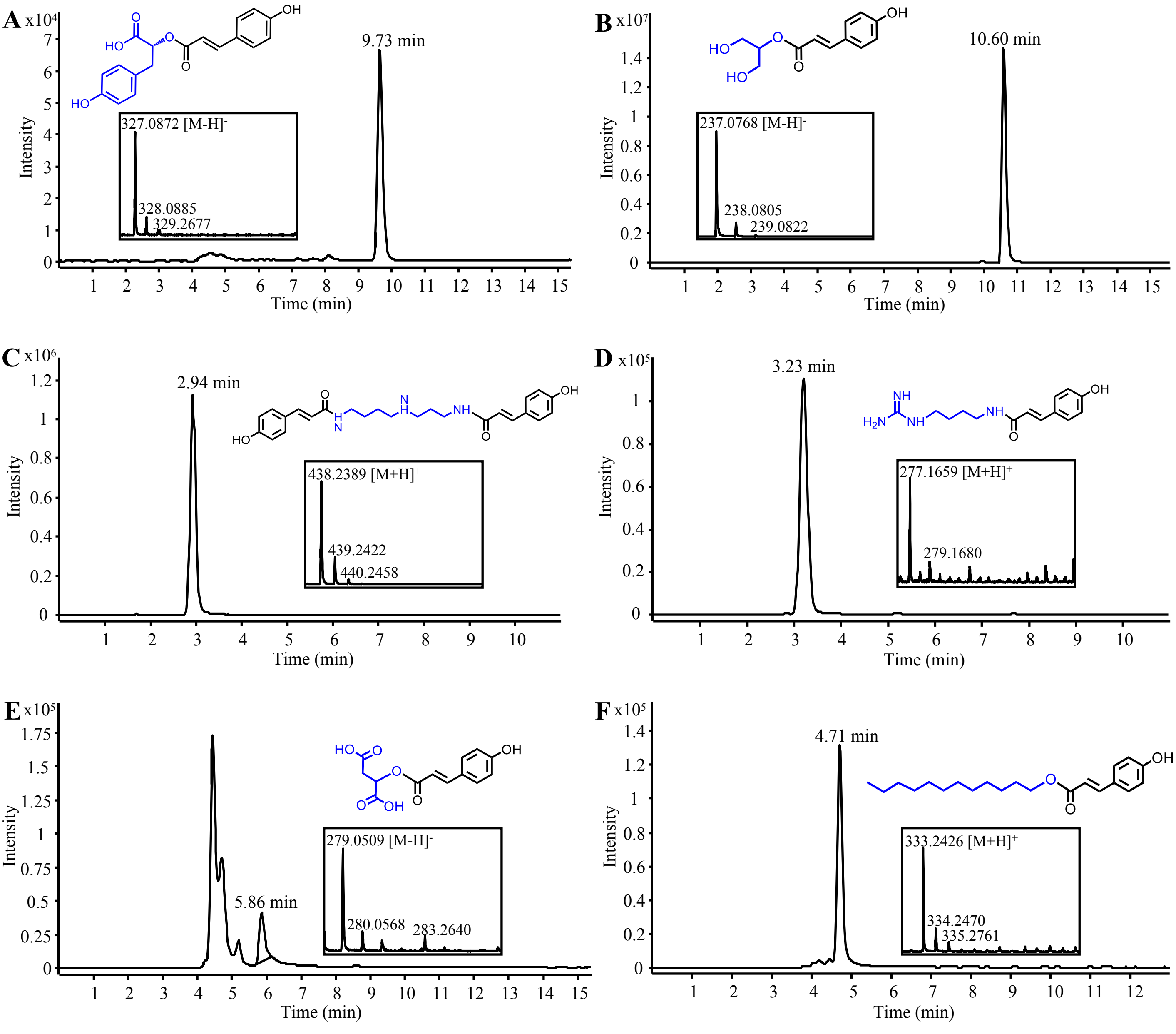

Fig. S1: Figure S1 Synthesis of *p*-coumarate esters and amides from tyrosine in yeast. *S. cerevisiae* strains co-expressing tyrosine ammonia-lyase FjTAL and 4CL5 with LaAT1 **(A)**, OsHCT4 **(B)**, AtSCT **(C)**, HvACT **(D)**, TpHCT2 **(E)** or AtHHT3 **(F)** were fed with 4-hydroxyphenyllactate **(A)**, nothing **(B, C)**, agmatine **(D)**, malate **(E)** or 1-dodecanol **(F)** for the synthesis of *p*-coumaroyl 4'-hydroxyphenyllactate **(A)**, *p*-coumaroyl glycerol **(B)**, N^1,N^8-disinapoyl spermidine **(C)**, *p*-coumaroyl agmatine **(D)**, *p*-coumaroyl malate **(E)** and dodecyl *p*-coumarate **(F)**, respectively.

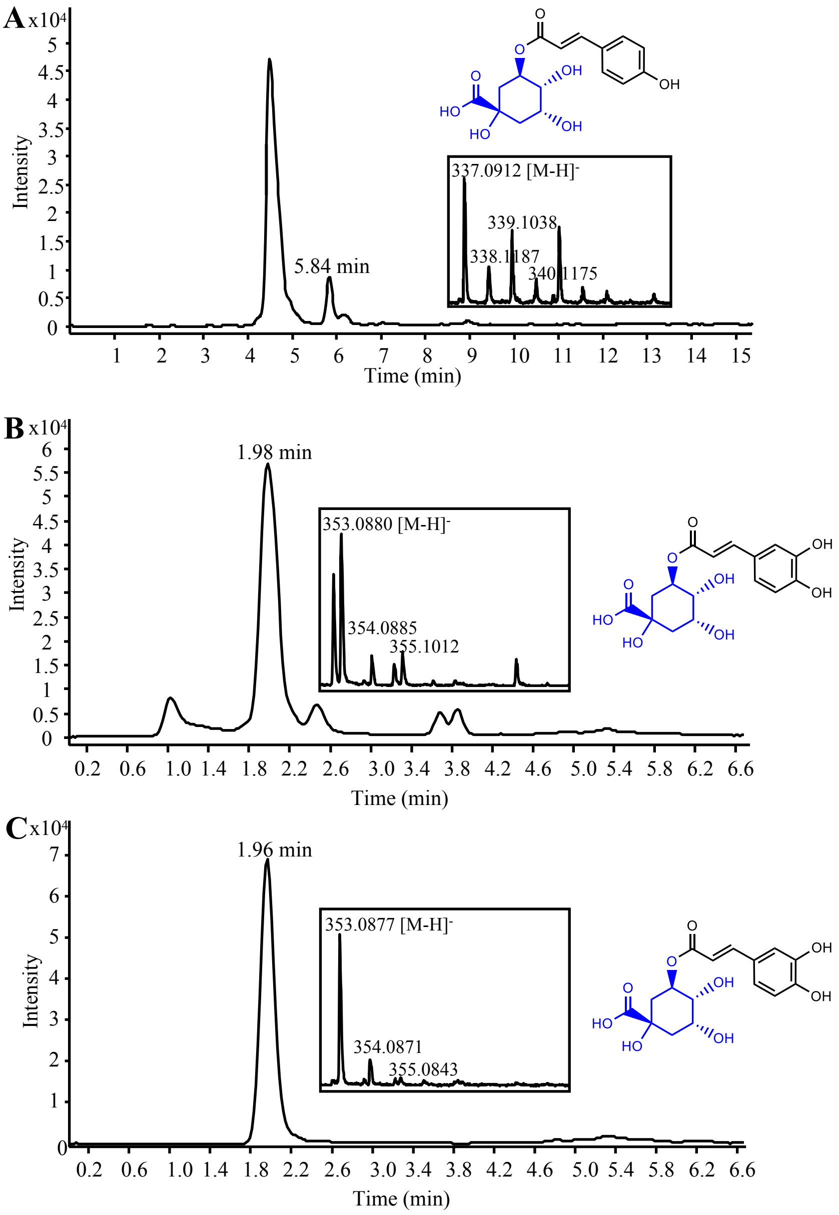

Fig. S2: Synthesis of quinate hydroxycinnamates in yeast. Representative LC-MS chromatograms obtained from analysis of the culture medium of a *S. cerevisiae* strain expressing 4CL5 and NtHQT are shown. The strain was fed with quinate and *p*-coumarate or caffeate for the synthesis of *p*-coumaroyl quinate **(A)** and chlorogenic acid **(B)**, respectively. The LC-MS chromatogram of a solution of authentic chlorogenic acid is also shown **(C)**.
